# Tuning Electrode Reactivity through Organometallic Complexes

**DOI:** 10.1021/acsami.3c01726

**Published:** 2023-06-09

**Authors:** Yi Shen, Yu Mu, Dunwei Wang, Chong Liu, Paula L. Diaconescu

**Affiliations:** † Department of Chemistry and Biochemistry, University of California, Los Angeles, Los Angeles, California 90095, United States; ‡ Department of Chemistry, Boston College, Chestnut Hill, Massachusetts 02467, United States

**Keywords:** functionalized electrode, surface functionalization, organometallic complex, electrode surface, switchable catalysis, electrocatalysis, electric field-assisted catalysis, integrated catalysis

## Abstract

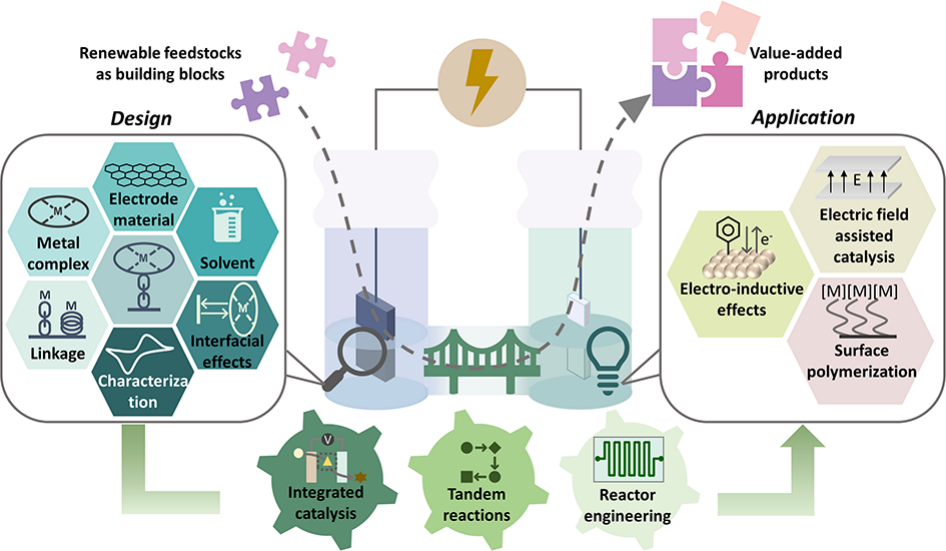

The use of molecularly modified electrodes in catalysis heralds a new paradigm in designing chemical transformations by allowing control of catalytic activity. Herein, we provide an overview of reported methods to develop electrodes functionalized with organometallic complexes and a summary of commonly used techniques for characterizing the electrode surface after immobilization. In addition, we highlight the implications of surface functionalization in catalysis to emphasize the key aspects that should be considered during the development and optimization of functionalized electrodes. Particularly, surface–molecule electronic coupling and electrostatic interactions within a hybrid system are discussed to present effective handles in tuning catalytic activity. We envision that this emerging type of hybrid catalytic system has the potential to combine the advantages of homogeneous catalysis and heterogeneous supports and could be applied to an expanded range of transformations beyond energy conversion.

## 1 Introduction

Over the past decade, electrocatalysis has been recognized as an accessible, scalable, and sustainable tool to address commonly encountered challenges in energy conversion and chemical synthesis.^1−4^ Recent developments in homogeneous electrocatalysis and electrode materials laid the foundation of a hybrid catalysis approach by providing numerous homogeneous and heterogeneous candidates to explore.^5^ Among those efforts, functionalizing electrodes with tunable molecular catalysts bridges the realms of heterogeneous and homogeneous catalysis and has become a viable alternative to traditional approaches in energy conversion and commodity chemical production.^6^ In pursuit of versatile, efficient, and selective catalysts, organometallic complexes mimicking highly efficient metalloenzymes have been brought under the spotlight for applications in electrochemical transformations.^7^

**Figure 1:**
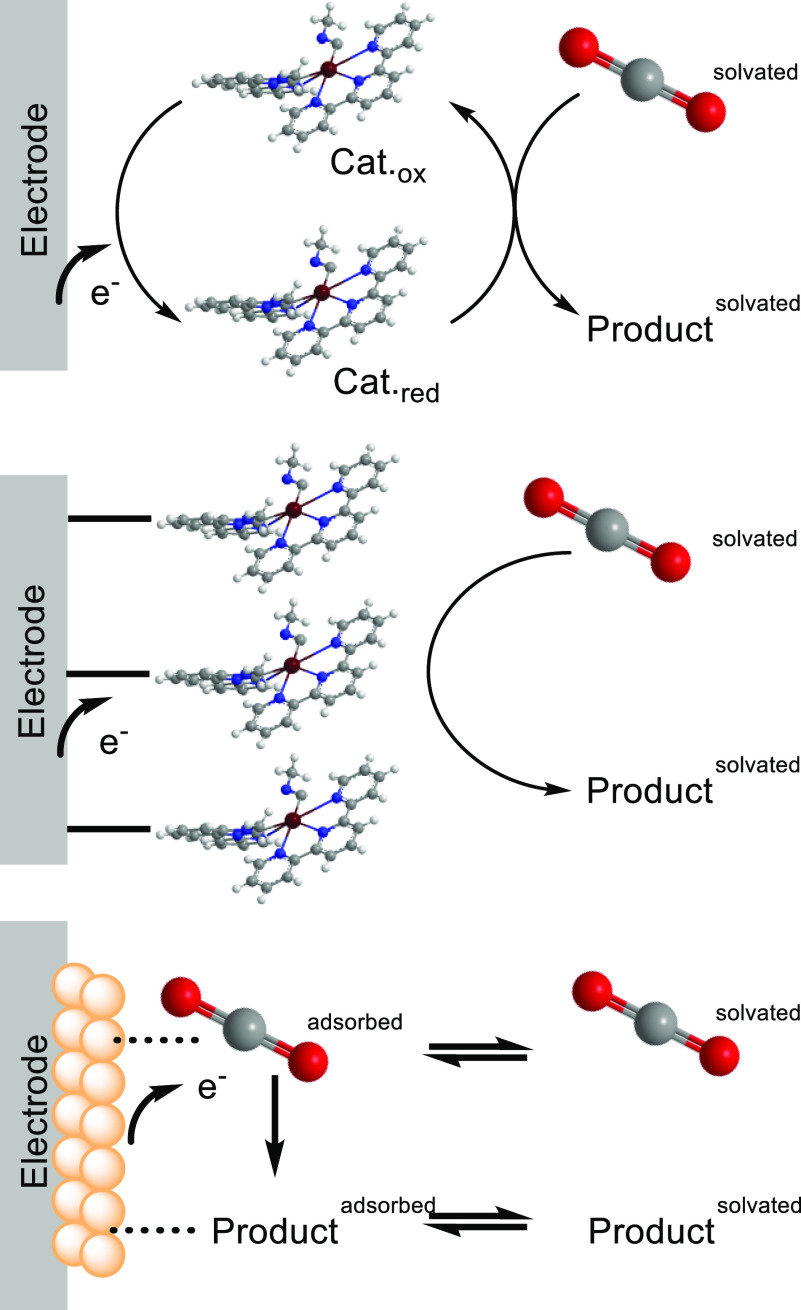
Illustration of catalytic CO_2_ reduction by a homogeneous (top), an immobilized (middle), or a heterogeneous (bottom) catalyst.^8^ Adapted with permission from ref (8). Copyright 2020 American Chemical Society.

The immobilization of molecular catalysts on an electrode surface may be a solution to bridge the gap between freely dispersed molecular compounds and heterogeneous catalysts (Figure 1). A molecular catalyst in electrochemistry has been previously identified to either homogeneously disperse in the solution or form active layers of molecules localized near the electrode surface.^9^ The most important distinction between heterogeneous vs molecular electrocatalysis is that active sites are well-defined molecules in the latter case.^10^ Given their versatility, tunability, and the possibility to understand their working mechanisms, homogeneous catalysts represented by organometallic complexes were introduced to electrochemical systems to increase accessibility and control reactivity.^11,12^ However, solvent incompatibility, slow diffusion rates, low electron transfer rates, and catalyst degradation remain hurdles on the road toward optimizing catalytic activity.^13^ On the other hand, heterogeneous catalysts, where electrodes directly participate in electron transfer, are promising for practical applications due to their robust and recyclable nature. It has been shown that immobilizing molecular catalysts onto an electrically addressable surface may help circumvent solvent incompatibility, in addition to promoting electron transfer, simplifying the separation process of products from the catalyst, and preventing the formation of aggregates.^11,13^ Thus, a surface-immobilized molecular catalyst is expected to be more efficient and recyclable in electrochemical synthesis than its nonimmobilized counterpart. An array of studies has demonstrated that chemically modified electrodes prepared by immobilizing organometallic complexes onto an electrically addressable surface provide an excellent platform for obtaining potent catalysts for key transformations related to energy generation.^7,13−21^

This review aims to stimulate ideas and critical thinking on the rational design of chemically modified electrodes through immobilizing a molecular functionality, primarily an organometallic complex, onto the electrode surface. Applications of the hybrid catalytic material focus on maximizing energy efficiency, improving catalyst lifetime, optimizing catalytic activity, and expanding application scope. By illustrating the unique reactivity and comparing the catalytic behavior of different hybrid systems, we strive to provide a systematic view of the field. First, we will offer a technical perspective on the possible approaches to immobilize a molecular catalyst based on the type of electrically addressable surface. Metal, metal oxide, and carbon-based electrode materials are discussed due to their broad application in the field of electrochemistry. Furthermore, strategies for surface immobilization of metal complexes *via* noncovalent and covalent interactions will be elaborated in detail for carbon-based electrodes given their accessibility, availability, and feasibility. Modifications of the molecular moiety may be required to accommodate the anchoring handles on the electrode surface in order to implement a specific linkage. We demonstrate the possibility to harness electrode-supported organometallic catalysts in electrochemical transformations *via* different immobilization methods and their disparate effects on catalytic activity. To provide a clear starting point for those who are new to the field, we also include techniques commonly employed in characterizing modified surfaces that play an indispensable role in interpreting the properties of the functionalized material. Aspects regarding the linkage, local reaction environment, and local structure of the designed system after immobilization will be discussed next along with applications of the functionalized electrode in electrocatalytic transformations related to energy conversion. Particular attention is devoted to the electrochemical control and tunability of the functionalized catalysts. Electronic and electrostatic interactions between the molecule–surface interface will be discussed in detail, showcasing necessary mechanistic investigations of the hybrid system. Local electric fields induced by charged functional groups of a supporting ligand represent another unique handle to modulate reactivity through electrostatic effects. Moreover, some compelling examples discussing the effect of externally applied electric fields in a hybrid system on catalytic turnover will be included to showcase the diversity of future applications. As this review will elaborate on the difference and connections between electrocatalysis and catalysis powered by electrostatic interactions, we hope to encourage efforts on broadening the scope of applications harnessing molecularly modified electrodes as potent catalysts. Overall, we aim to inspire researchers to interface organometallic chemistry, surface chemistry, and electrochemistry.

## 2 Types of Surfaces

In this section, we will introduce common strategies to construct a molecularly modified electrode or the *hybrid* catalytic material based on the type of electrode surface. Depending on available anchoring sites and immobilization methods, modifications of electrically addressable surfaces (metals, metal oxides, and carbon-based electrodes) with anchoring handles may be necessary. A representative selection of examples concentrating on immobilizing organometallic complexes on each type of electrode surface will be discussed.

### 2.1 Metal and Metal Oxide Electrode Surfaces

**Figure 2:**
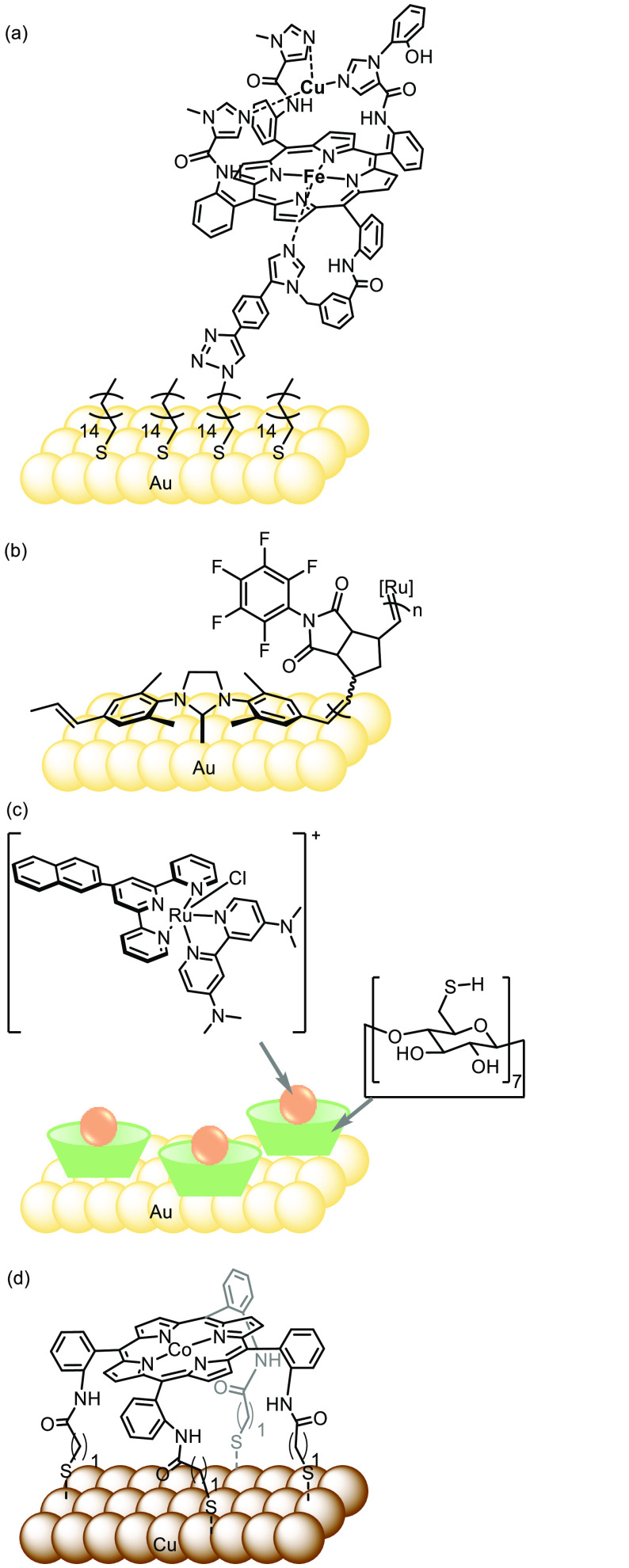
(a) Covalently attached functional model of cytochrome c oxidase to a gold electrode by using a terminal acetylene with a terminal azide bound to a self-assembled monolayer of thiolates.^26^ Adapted with permission from ref (26). Copyright 2007 American Association for the Advancement of Science. (b) Gold surface modified with N-heterocyclic carbenes with polymer brushes containing ruthenium.^30^ Adapted with permission from ref (30). Copyright 2013 American Chemical Society. (c) Ruthenium complex immobilized on a gold electrode surface through a surface-bound thiolated cyclodextrin host.^33^ Adapted with permission from ref (33). Copyright 2021 Nature Publishing Group. (d) Copper electrode surface functionalized with a cobalt porphyrin supramolecular cage.^34^ Adapted with permission from ref (34). Copyright 2017 American Chemical Society.

Metal electrodes such as Cu, Au, and Ag play an essential role in electrochemical studies.^22^ The intrinsic electronic structure of the metal electrode determines the electrocatalytic activity of the system, while the desorption energy of active species on the surface can be modulated by polarizing the electrode surface.^23^ In light of that, surface modification of metal electrodes was envisaged to modulate the catalytic activity of the anchored species. For example, Deng and co-workers triggered an electropolymerization of molecules to generate organic frameworks on the surface of a gold electrode that created a compact interface enriched by amino groups.^24^ This noncovalent modification of the metal electrode surface resulted in higher CO production and selectivity than a bare gold electrode under the same conditions. To increase the available anchoring sites on metal surfaces, premodifications of the metal are commonly required. Sulfur-based self-assembled monolayers on gold are pervasive.^25^ For example, a gold surface modified with alkanethiols featuring terminal azides was functionalized by a biomimetic metal complex equipped with terminal alkynes *via* a “click reaction” (Figure 2a).^26^

*N*-heterocyclic carbenes (NHCs) show great stability in binding to metal electrodes, especially gold electrodes, with versatility for further functionalization.^27−31^ Adjustable polymer brushes bound to ruthenium were successfully attached to a gold surface through an NHC–Au interaction (Figure 2b).^30^ A tutorial review highlighting recent advances in functionalizing gold surfaces has been published by Engel et al.^32^

“Host–guest” interactions represent a noncovalent approach toward localizing metal complexes on the metal electrode surface. Sévery and co-workers reported the binding of a molecular catalyst to a metal electrode surface through host–guest complexation with surface-attached cyclodextrins (Figure 2c).^33^ This approach displayed a surprisingly stable immobilization under catalytic conditions, representing a proof of concept for employing a surface host to immobilize an organometallic complex.

Copper represents an alternative electrode material to expensive gold. Chang and co-workers directly anchored supramolecular cages of cobalt porphyrins on a copper electrode surface *via* a Cu–S bond formed by a thiolated porphyrin affording a molecular–material interfacial catalyst for electrochemical reduction of CO to value-added multicarbon products (Figure 2d).^34^ An order of magnitude improvement in selectivity and activity was achieved by the functionalized electrode in comparison to the bare copper electrode surface. However, it is worth noting that metal surfaces tend to participate in redox reactions, which cannot be fully disentangled from the activity of the immobilized metal complexes.

Metal oxides represent another promising platform for immobilization because of their accessibility, thermodynamic stability, and potential to be prepared in various nanometer-sized structures.^35^ Metal oxides have been extensively employed as solid supports for molecular complexes.^35−38^ In addition, metal oxides display tunable optical and electrical properties (e.g., transparent and electrically conductive indium tin oxide, fluorine-doped tin oxide, and semiconductive titanium oxide), making them great candidates as electrode materials.^39−47^

Organometallic complexes can be introduced onto metal oxide supports either by direct bonding or *via* intervening ligands.^48^ Direct adsorption onto metal oxide surfaces and physical entrapment of molecules inside porous layers are common strategies to assemble such heterogenized catalysts for transformations such as water oxidation.^49,50^ It is important to note that the metal/ligand can react with the oxide support in various ways. Surface hydroxyl groups were found to serve as both Brønsted acid and base sites.^48^ However, the focus of this review is not the coordination chemistry or reaction mechanisms of supported metal complexes on metal oxide surfaces; instead, we highlight the possibilities to leverage the interaction with metal oxide surface hydroxyl groups.^48,50^

**Figure 3:**
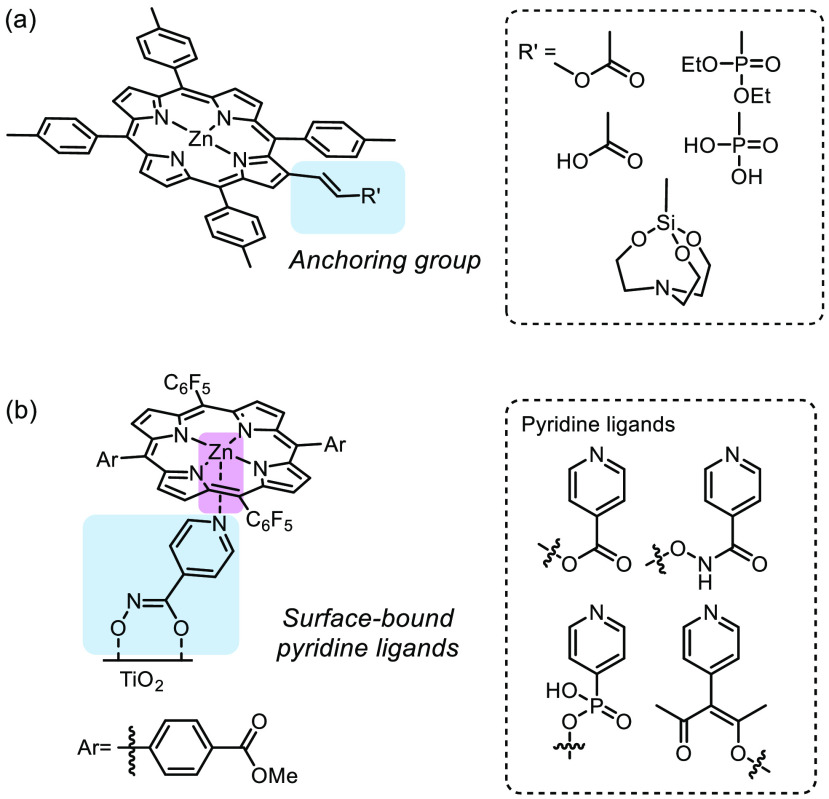
(a) Examples of metal complexes with molecular modifications on the ligand structure to enable surface anchoring.^57^ Adapted with permission from ref (57). Copyright 2013 The Royal Society of Chemistry. (b) Metal complexes coordinated to surface-bound pyridine ligands as linkers.^62^ Adapted with permission from ref (62). Copyright 2013 American Chemical Society.

Axial coordination of metal centers to the abundant hydroxyl groups on metal oxide surfaces is readily available.^15,38,40,51,52^ Moreover, various types of anchoring handles were developed to create diverse and stable linkages between the surface and metal complex.^35−37,53−56^ Metal complexes can be immobilized covalently onto metal oxide surfaces by implementing a designed anchoring handle in the ligand structure.^15,41,43,57−61^Figure 3a depicts an example where anchoring groups such as carboxylic ester/acid, phosphonic ester/acid, and silatrane are incorporated into the ligand of a zinc complex for binding to the metal oxide surface.^57^ On the other hand, metal oxide surfaces can be modified with those anchoring groups which serve as the surface ligand to coordinate with the metal center.^35,37,62^Figure 3b showcases a zinc complex binding to a surface-bound layer of linkers *via* metal coordination.^62^ Other examples of anchoring handles include π–π interactions between compounds bearing aromatic functionalities and pyrene-modified metal oxide surfaces,^42,47^ as well as covalent grafting of metal complexes containing vinyl functionalities to surface hydroxyls by electro- or light-initiated polymerizations.^36,40,41,43,49,54−56,60,63−66^

**Figure 4:**
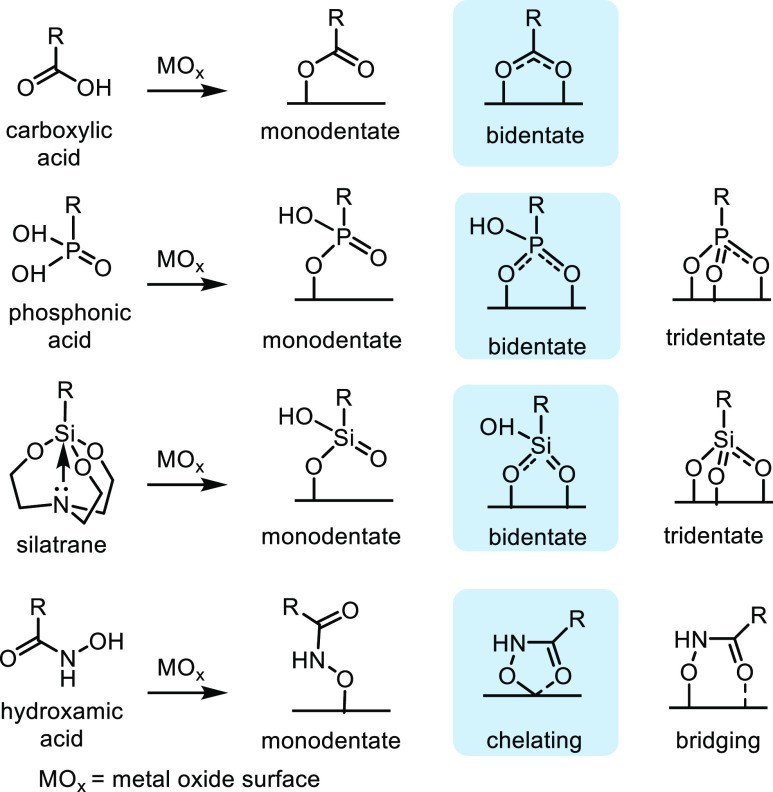
Possible binding modes of carboxylic acid, phosphonic acid, silatrane, and hydroxamic acid surface anchors to metal oxide surfaces (MO_*x*_). The most favored binding modes are highlighted.^37^ Bridging refers to binding of two metal ions on the metal oxide surface, and chelating refers to the binding of a chelating ligand to a single metal. Adapted with permission from ref (37). Copyright 2017 The Royal Society of Chemistry.

Common anchoring groups exemplified by oxyacids (e.g., carboxylic acids, phosphonic acids, and hydroxamic acid) and silatranes display different binding modes when attached to the metal oxide (Figure 4). The type of binding could be analyzed by infrared spectroscopy using the existence/disappearance or change in peak intensity of infrared active bonds, such as C=O stretching.^57^ The presence of various types of binding modes to the surface hydroxyl groups and the possible coexistence of multiple binding modes differentiates metal oxide supports from metal and carbon-based surfaces, as will be discussed later in the review.

### 2.2 Carbon-Based Electrode Surfaces

**Figure 5:**
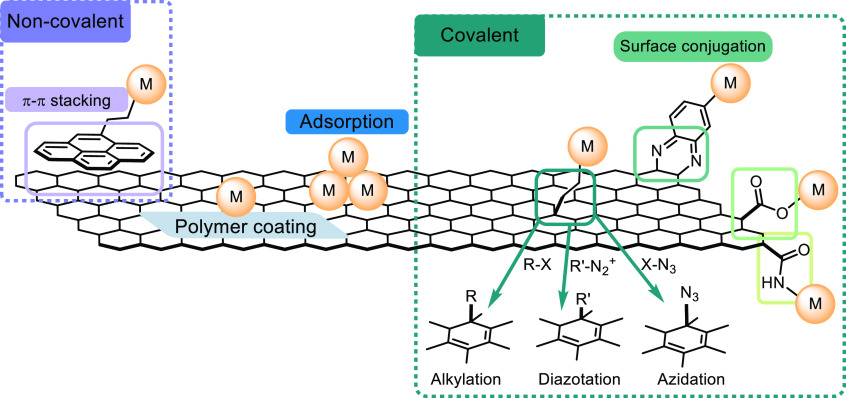
Common strategies for molecular surface functionalization of carbon-based electrodes. Pretreating the surface may be necessary to provide available sites for functionalization (e.g., oxidized glassy carbon electrode to increase carboxylic functionalities for the potential formation of ester or amide linkage).^11,72−76,97−99^ Chemical structure of carbon electrode is simplified and does not reflect its complex chemical nature. Adapted with permission from ref (72). Copyright 2019 American Chemical Society.

Carbon-based electrodes feature a high electrical conductivity, high surface area, tunable porous structure, and low cost, making them an attractive solid support to manipulate.^67−69^ Various immobilization methods on carbon-based electrodes (e.g., graphene and carbon nanotubes) have been reported.^13^ Molecular modifications of carbon-based electrodes combine the stability and conductivity of carbon electrodes with the high activity and selectivity of metal complexes.^11,70,71^ Common strategies for functionalizing carbon-based electrodes include polymer coating, adsorption, and noncovalent and covalent methods (Figure 5).^11,72−76^ Among those methods, noncovalent and covalent methods for direct surface functionalization give rise to relatively strong interactions/rigid bonds of various type and could be widely adapted to an array of structurally diverse molecular components. Noncovalent methods involve nonbonding interactions such as van der Waals forces, hydrogen bonding, and π–π stacking interactions. Among these interactions, π–π stacking between the supporting ligand of a metal complex and the electrode represents a straightforward strategy of immobilizing aromatic compounds on a carbon-based electrode without a tremendous amount of work to modify the surface (Figure 5).^13,77−80^ Applications of immobilized molecular electrocatalysts *via* noncovalent methods in reactions related to energy conversions such as CO_2_ reduction and oxygen evolution reaction have been well-studied and summarized.^13,79−85^ Despite the convenience of the noncovalent immobilization method, catalyst leaching, and aggregation still need to be addressed to optimize the durability and activity of the electrocatalytic system.

Covalent immobilization tends to be more complicated than noncovalent methods but usually provides a robust connection through between the surface and the molecular catalyst. The complexity of the true chemical nature of the carbon electrode surface adds to complications and challenges when using covalent immobilization. Both electrochemical and chemical reactions, e.g., electrochemical reduction of diazonium^86−88^ and alkyne–azide cycloaddition,^89−91^ could be utilized to create covalent bonds between an electrode and the surface attachment (Figure 5).^13^ It is also possible to functionalize the surface through other covalent bonds such as forming esters or amides. Pretreating the electrode surface to increase the number of bonding sites is necessary for implementing less common interactions, such as *o*-phenylenediamines/*o*-quinones condensation, where *o*-quinones are naturally less available.^20,21,92−96^ Detailed discussions on how to create each type of linkage will be presented in the next section.

Key factors to assess when selecting an appropriate electrode material for a specific application include conductivity, stability, morphology, and versatility of the electrode material. Materials with high electrical conductivity, such as gold or platinum, ensure an efficient electron transfer process that can lead to an enhanced electrochemical activity. A robust support mitigates the degradation of the electrode surface during electrochemical reactions. In addition, the morphology of the solid substrate may influence the electrochemical behavior of the modified electrode. For instance, a porous electrode material with a high surface area provides an increased number of sites for molecular modification and, yet, might result in encapsulating the active species within the pores.^100^ Importantly, the electrode material has to accommodate the possible modifications of the molecular catalyst to provide a feasible platform addressing the hybrid strategy. In the next section, we will discuss possible modifications to the molecular catalyst that provide necessary molecular handles and create different linkages with the electrode support. Due to the breadth of this field, we will concentrate on carbon-based electrode surfaces as they feature a highly conductive, relatively inexpensive, and easily modifiable material to functionalize for broad applications.

## 3 Types of Immobilization Strategies

The general strategy of developing a molecularly modified electrode system involves the selection of a catalyst that is highly active in the homogeneous state, followed by configuring methods to anchor it onto the electrode surface. In the previous section, we discussed common strategies employed for electrode surface functionalization of carbon-based electrodes as depicted in Figure 5. However, the molecular compound of interest may not have the necessary functional group to form a designated handle for surface functionalization. In this context, molecular modifications of the ligand structure are necessary. Common linkages will be elaborated based on noncovalent versus covalent interactions between the surface and appended metal complex. To showcase the benefits of different immobilization strategies, we will also briefly discuss how the proximate electrodes functionalized with organometallic complexes exert their influence in catalysis.

### 3.1 Noncovalent Immobilization of Metal Complexes

**Figure 6:**
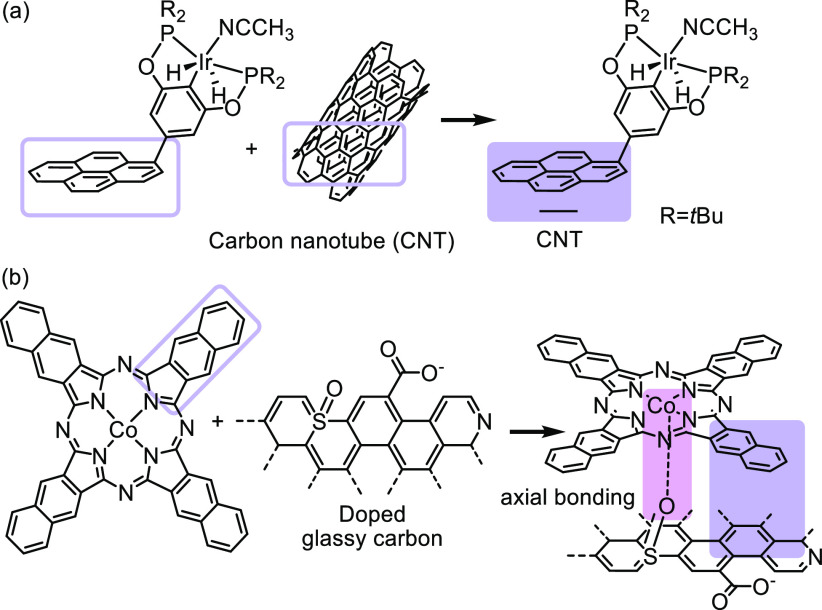
Noncovalent immobilization of metal complexes onto a carbon-based electrode. (a) Illustration of surface-bound Ir pincer catalyst.^102^ Adapted with permission from ref (102). Copyright 2014 John Wiley & Sons, Inc. (b) Immobilized cobalt naphthalocyanine *via* π–π stacking and axial bonding.^105^ Adapted with permission from ref (105). Copyright 2019 John Wiley & Sons, Inc.

Carbon-based electrodes represent ideal candidates for noncovalent immobilization of metal complexes because of their high surface area, stability, and, most importantly, availability to form π–π interactions with an aryl group of the ligand framework.^68,101^Figure 6a depicts the incorporation of pyrene on an iridium pincer complex to introduce additional π–π interactions.^102^ Carbon nanotubes (CNTs) are commonly used as the heterogeneous substrate; however, metal oxides are also suitable for this method.^42,47^ Increased catalyst lifetime, enhanced activity, and/or improved product selectivity of the immobilized catalyst are commonly observed compared to the parent metal complex in electrocatalytic reactions, likely due to the efficient electron transfer between the electrode surface and the immobilized metal complex *via* π–π stacking.^80,83,102−105^ For example, the iridium complex immobilized on CNT displayed high turnover numbers (TON = 54,000) and turnover frequencies (TOF = 15 s^–1^) in the selective electrocatalytic reduction of CO_2_ to formate.^102^

Metal complexes supported by ligands such as porphyrins and phthalocyanines and their derivatives also suit the needs for noncovalent immobilization *via* π–π stacking.^13,42,47,80,102−105^ Axial coordination of the metal center to the heteroatoms doped on the carbon electrode offers additional promoting effects after immobilization (Figure 6b). Upon deposition onto a doped glassy carbon electrode, the cobalt naphthalocyanine complex resulted in up to 97% Faradaic efficiency in CO_2_ to CO reduction.^105^ By contrast, the molecular cobalt complex used in solution was inactive toward CO_2_ reduction, indicating novel reactivity induced by surface functionalization.

### 3.2 Covalent Immobilization of Metal Complexes

#### 3.2.1 Covalent Linkage by the Reduction of Diazonium Salts

**Figure 7:**
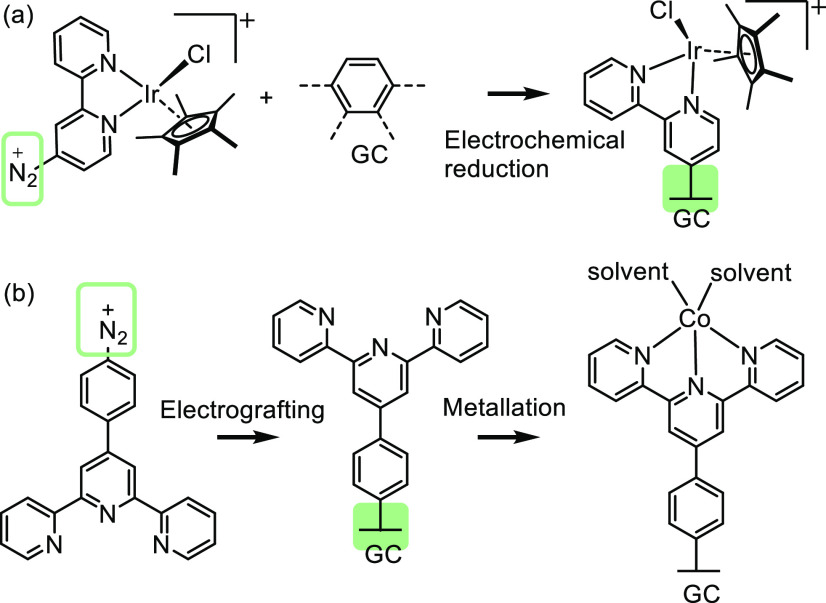
(a) Electrografting of iridium complex for water oxidation.^108^ Adapted with permission from ref (108). Copyright 2012 American Chemical Society. (b) Electrografting of terpyridine followed by metalation to obtain surface-bound cobalt complex.^110^ Adapted with permission from ref (110). Copyright 2015 The Royal Society of Chemistry.

Electrochemical and photochemical reductions of diazonium salts have been widely deployed in functionalizing metal, carbon, and semiconductor surfaces.^63,87,88,106−110^ Grafting of diazonium salts, derived from amine groups, has been widely applied for creating a covalent linkage between a metal complex with a carbon-based electrode.^109−112^ An enhancement in catalytic activity and a significant improvement of catalyst life are commonly observed in the hybrid system obtained by this method. For example, iridium complexes grafted onto carbon electrodes achieved a turnover frequency of up to 3.3 s^–1^ and a turnover number of 644 during the first hour of the electrochemical water oxidation reaction (Figure 7a),^108^ showing an enhanced activity compared to the molecular analogue that only reached a turnover number of ∼150 before being deactivated.

In another example, terpyridine was modified with diazonium to be electrochemically reduced for grafting onto the electrode.^110^ Followed by metalation with cobalt or nickel, a metal complex functionalized electrode was obtained and tested in electrocatalytic transformations (Figure 7b). The modified system displayed incredible robustness and activity in proton reduction, however, showing modest activity and limited stability in CO_2_ reduction.

In the examples illustrated in Figure 7, the electrochemical reduction of diazonium salts enables the formation of robust linkages between the surface and the attachment, affording active immobilized organometallic complexes in electrocatalytic reactions, with a prolonged lifetime and an improved performance. However, electrochemical grafting requires the use of a potentiostat to reduce a diazonium-modified molecular species, limiting the versatility of the method in some cases when unstable compounds are used. In that case, photochemical grafting can be used as an alternative method.^88^

#### 3.2.2 Covalent Linkage *via* a Click Reaction

**Figure 8:**
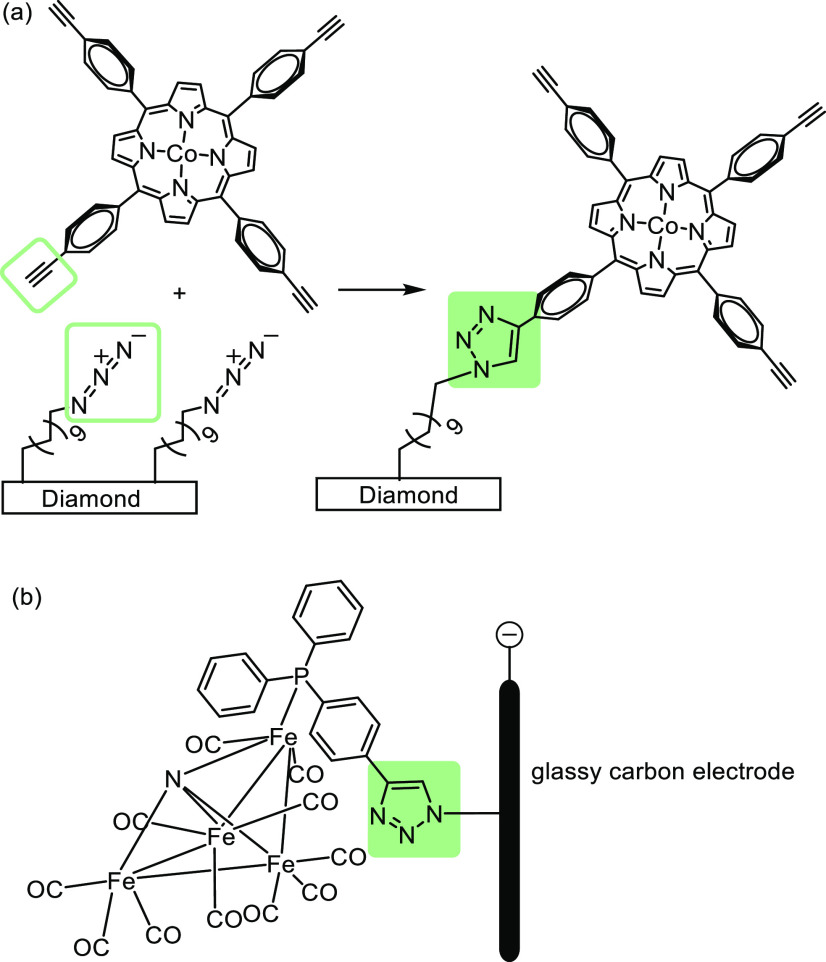
(a) Covalent immobilization using a click reaction with a cobalt complex.^17^ Adapted with permission from ref (17). Copyright 2012 American Chemical Society (b) Covalent immobilization of an iron complex *via* “click” reaction.^113^ Adapted with permission from ref (113). Copyright 2019 American Chemical Society.

Copper-catalyzed azide–alkyne cycloaddition, one of the click reactions, represents another method to introduce a rigid covalent linkage between the surface and the anchored compound.^66,90,91^ Anchoring a cobalt complex onto a doped conductive diamond electrode *via* a click reaction afforded a modified electrode material with good stability and activity in CO_2_ reduction (Figure 8a).^17^ The catalytic activity of the immobilized system remained unchanged after 1000 cycles, indicating superior stability. The same immobilization strategy was also applied to other metal complexes such as [Fe_4_N(CO)_12_]^−^ (Figure 8b).^113^ Remarkably, the iron complex modified electrode maintained its activity in CO_2_ reduction after 3 days of testing, indicating a significant improvement in catalytic lifetime compared to the homogeneous analogue. Interestingly, mechanistic changes were observed upon immobilization: the homogeneous compound underwent stepwise electron and proton transfer, whereas the heterogenized system went through a concerted electron–proton transfer, implying electronic coupling between the anchored compound and the electrode.

#### 3.2.3 Covalent Linkage with Amino-Substituted Ligands

**Figure 9:**
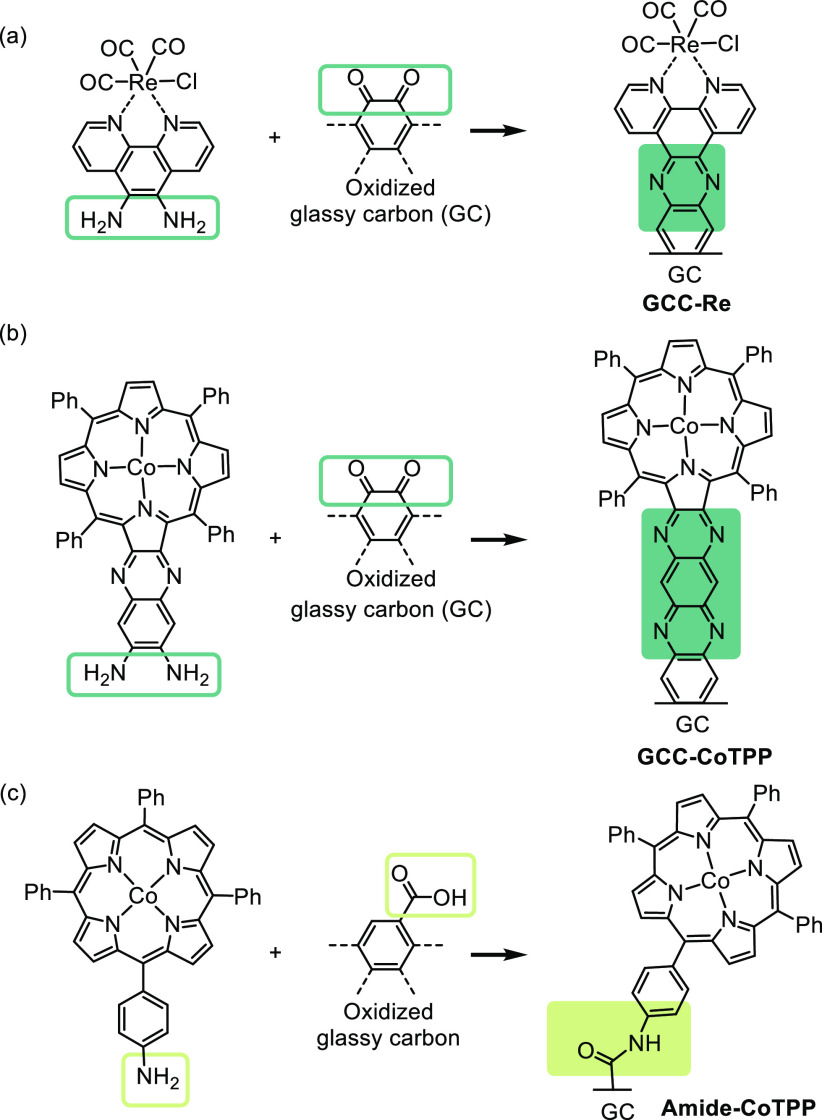
(a) Representation of graphite-conjugated rhenium active site for CO_2_ reduction.^96^ Adapted with permission from ref (96). Copyright 2016 American Chemical Society. (b and c) Immobilization of cobalt porphyrin on a modified electrode surface through a conjugated pyrazine and an amide linkage, respectively.^20^ Adapted with permission from ref (20). Copyright 2019 American Chemical Society.

Appended amines on the ligand of an organometallic complex may be subject to covalent linkage *via* amide bond formation and diamine condensation.^20,21,92−96,114^ The presence of carboxylic acids and *o*-quinones on the edge plane of graphitic electrodes provides available sites for forming amide bonds and fully conjugated pyrazine linkages. Re(5,6-diamino-1,10-phenanthroline)(CO)_3_Cl was fully conjugated to graphite and achieved superior activity in CO_2_ reduction (Figure 9a).^96^ The graphite-conjugated Re catalyst (GCC-Re) displayed remarkably high turnover numbers, greater than 12,000, with CO as the only product. A graphite-conjugated cobalt tetraphenylporphyrin (GCC-CoTPP) and a nonconjugated cobalt porphyrin complex (Amide-CoTPP) were investigated in comparison (Figure 9b,c).^20^ A dramatic enhancement in the rate of oxygen reduction was observed in GCC-CoTPP compared to Amide-CoTPP.^20^ It is speculated the highly conjugated pyrazine linkage enabled a rapid electron transfer, which significantly enhanced the catalytic activity. Detailed investigations of the efficient electronic communication enabled by the graphite-conjugated system will be elaborated in Section 5.

**Figure 10:**
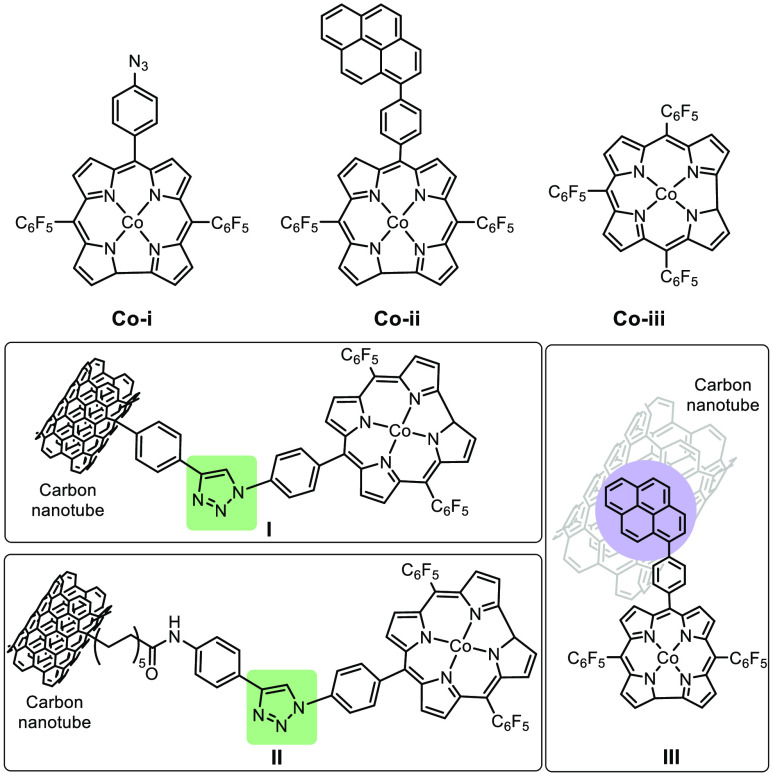
Homogenous cobalt complexes functionalized on carbon nanotubes affording modified electrodes through a short-conjugated linker (**I**), long alkene linker (**II**), and π–π interactions (**III**).^115^ Hybrid electrode catalysts **I** and **II** are made from homogeneous cobalt complex **Co-i**. **III** is made from **Co-ii**. A cobalt complex without anchoring handles is exemplified by **Co-iii**. Adapted with permission from ref (115). Copyright 2018 John Wiley & Sons, Inc.

It is important to keep in mind that one type of immobilization may not be necessarily superior to another method. A study comparing functionalized electrodes through a short-conjugated linker (**I**), a long alkene linker (**II**), π–π interactions (**III**), and simple adsorption (**IV**) of cobalt complexes demonstrated that different types of immobilization methods may be advantageous and preferable under specific conditions (Figure 10).^115^ Experimental results on such functionalized carbon nanotubes in electrocatalytic hydrogen and oxygen evolution revealed that the short-conjugated linkage surpassed π–π interactions and the long alkene linker in electron transfer ability, and a similar trend was observed on catalytic activity.^115^ In contrast to the activity trend observed for the hybrid systems (**I** > **III** > **II** > **IV**), homogeneous compounds displayed a different trend (**Co-iii** > **Co-ii** > **Co-i**). Overall, all three immobilization methods had the potential to afford a catalytically active material, which was found to have an improved lifetime and minimal catalyst aggregation. Thus, an evaluation of the advantages and disadvantages of different immobilization strategies and a comparison study of the resulted systems is necessary for reaction optimization.

To summarize, a typical design of a molecularly modified electrode generally encompasses a molecular catalyst; in this case, an organometallic compound, with optimal performance for a specific type of reaction, and an electrode support as the electron source.^114^ However, such a design assumes that the activity of the molecular catalyst translates invariably after heterogenization. In reality, ligand structure modifications can change the catalytic behavior of the selected organometallic compound dramatically. Therefore, a comprehensive study of the modified metal complex before surface immobilization is necessary. Characterization of the surface after functionalization also helps provide an understanding of the hybrid system and ensures the desired catalytic activity. Characterization methods will be discussed in Section 4.

Second, because of the disparity among different approaches with respect to linkage stability, versatility, and feasibility, some immobilization methods may not be suitable for a particular type of metal complex, as the conditions required to carry out the functionalization might be too harsh for the metal complex to survive. In this regard, combining a relatively stable metal complex with compelling catalytic activity with a practical immobilization method could save a lot of effort. Alternatively, a pro-ligand with an appropriate molecular handle can be immobilized followed by metalation (Figure 7b).^109,110^ In this scenario, however, the direct adsorption of metal ions onto the surface might be inevitable.

**Figure 11:**
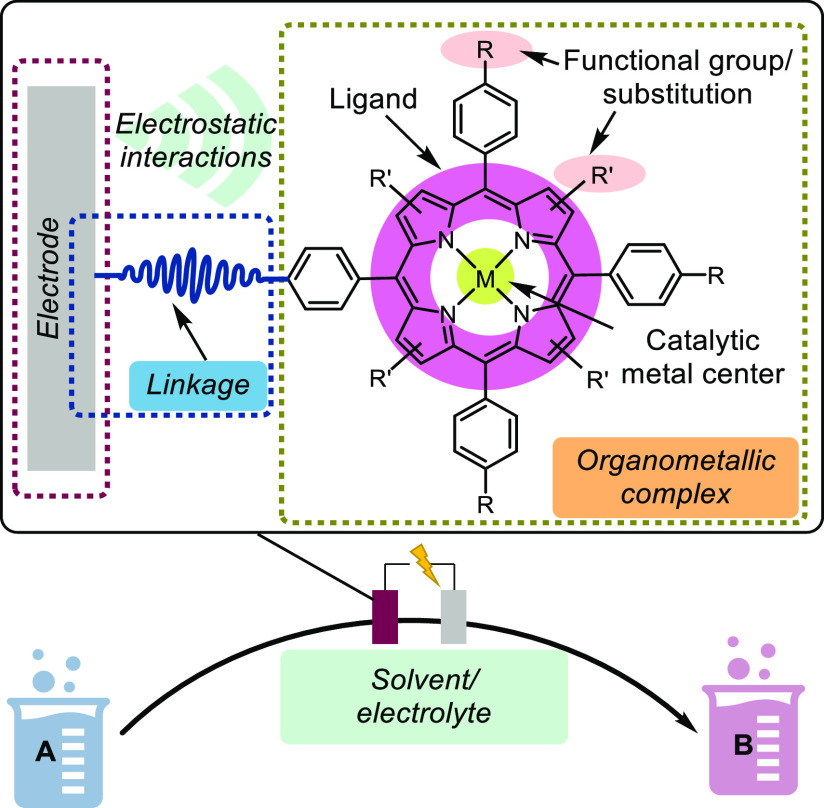
Molecularly modified electrode by anchoring a catalytically active organometallic complex and components to consider for an optimal design.^13^ Adapted with permission from ref (13). Copyright 2020 The Royal Society of Chemistry.

Lastly, surface immobilization may induce molecule–surface interactions and interactions between molecular entities that may lead to different implications. Catalyst loading and aggregation, solvent and electrolytes, and many other factors have to be considered, in addition to the choice of electrode and linkage for a hybrid catalytic material in real life applications. We include the aspects to consider for a rational design of an electrocatalytic system enabled by a molecularly modified electrode in Figure 11 such as electronic communication between the modified ligand and the electrode surface impacted by the immobilization *via* molecular linkage (electronic coupling), electrostatic interfacial interactions in the electrolyte, effects of functional groups on the ligand, and possible metal–metal interactions upon surface depositing. In Section 5, we will discuss specific applications of functionalized electrodes.

## 4 Characterization Methods of Heterogenized Systems

A comprehensive characterization of the molecularly modified surface paves the way to apply the functionalized material in catalysis. Most organometallic complexes with redox-active metal centers or redox noninnocent functionalities can deliver electrochemical responses in cyclic voltammetry to display unique features. Besides electrochemical characterization, heavy elements and representative binding modes with the electrode surface can also be analyzed by spectroscopic techniques such as UV–vis, IR, and X-ray photoelectron spectroscopy, which could provide complementary information to cyclic voltammetry under some circumstances.^38,51^

### 4.1 Cyclic Voltammetry

**Figure 12:**
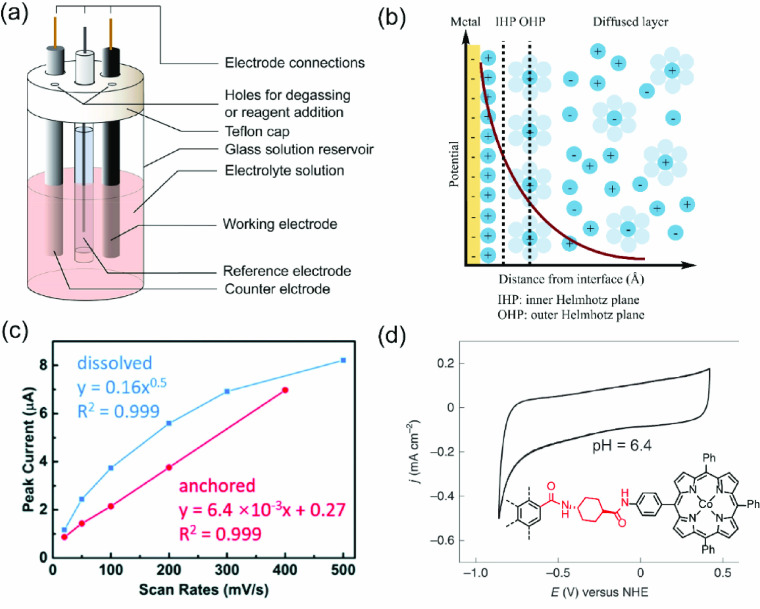
(a) Illustration of a three-electrode system for cyclic voltammetry measurements.^116^ Reproduced with permission from ref (116). Copyright 2017 American Chemical Society. (b) Gouy–Chapman–Stern model at the metal–electrolyte interface.^117^ Adapted with permission from ref (117). Copyright 2022 American Chemical Society. (c) Relationship between peak currents and scan rates for dissolved and anchored active species.^40^ Reproduced with permission from ref (40). Copyright 2021 The Royal Society of Chemistry. (d) CV trace of redox-active species strongly coupled with electrode surfaces.^114^ Reproduced with permission from ref (114). Copyright 2022 Nature Publishing Group.

The experiment of collecting the reversible behavior between the flowing current and a linearly changed potential with time is generally known as cyclic voltammetry (CV). Cyclic voltammetry is commonly carried out in a three-electrode system (Figure 12a).^116^ In such a system, the working electrode will host the redox reaction of interest and the counter electrode can balance the current flowing from the Faradaic reaction on the working electrode surface. The reference electrode will allow accurate measurements of the working electrode potential. In the three-electrode system, the Gouy–Chapman–Stern model at the interface between the working electrode and electrolyte is often used to describe the interfacial effect (Figure 12b).^117^ The double layer on an electrode surface includes a compact double layer within the outer Helmholtz plane (OHP) and a diffusion layer connected to ion redistribution.^118^ To ensure a negligible contribution from the migration to the mass transport of the charged active species during a Faradaic reaction, an excess of supporting electrolyte is usually used to simplify the mathematical treatment of the Faradaic current and establish a uniform ionic strength throughout the solution even under steady-state Faradaic conditions.^119^

Cyclic voltammetry is a powerful tool to characterize the electrode surface modified with molecular functionalities.^21,39,40,42,114^ Unlike their solubilized counterparts that are free to move around in an electrolyte solution, surface-anchored compounds have a limited diffusion range depending on the linkage length and solvent, and usually do not diffuse through the electrical double layer. They gain or lose electrons through electron transfer due to the directional potential difference with the electrode. Therefore, the peak current of surface anchored compounds in typical cyclic voltammograms would be linearly proportional to the scan rate rather than the square root of the scan rate as for their free soluble analogues,^120^ a feature that could be employed to validate that the compounds are anchored (Figure 12c).^40^ Furthermore, the symmetry of the *i*–*E* trace (i.e., the relationship between current and voltage during scanning) can reflect the system’s chemical stability (i.e., surface anchored compounds do not decompose or get dissolved) and electrochemical reversibility (i.e., Nernstian wave).

Two parameters may be extracted from the analysis. One is the ratio between the cathodic and anodic peak current (i.e., *i*_pa_/*i*_pc_), and the other is the separation of the two peak potentials (i.e., *E*_pa_ – *E*_pc_). For a chemically stable system, the ratio of peak currents is generally equal to 1.^120^ However, one should be cautious in calculating it because the anodic peak current (if the cathodic scan is started first) must be corrected to a zero current baseline to mitigate the disturbance of the capacitance effect at the interface.^120^ For a Nernstian wave with a one-electron transfer, the difference between the two peak potentials should be 57–60 mV at room temperature.^119^

Cyclic voltammetry may not be helpful when characterizing some noncovalently anchored systems with strong electronic interactions between the electrode and the molecular compound.^114^ Under such a scenario, the Fermi level of the electrode couples strongly with the energy level of the redox center in the anchored system, causing no potential drop between the electrode and the molecular compound (specific examples will be discussed in Sections 5.1 and 5.2).^121^ Therefore, cyclic voltammetry can only reflect the Faradaic reaction of the supporting electrolyte. Figure 12d exemplifies the current limit of obtaining a comprehensive picture of the carbon electrode. The different possible electronic interactions,^121^ as well as the complex chemical structure of carbon electrode surfaces, pose significant challenges in characterizing the corresponding active sites, either covalently or noncovalently anchored. Two approaches can address such challenges: (1) additional features from the cyclic voltammogram, such as the peak width and its dependence on different scan rates should be evaluated and the whole electroanalysis should include results at different experimental conditions; (2) the data interpretation from cyclic voltammetry should be corroborated with other surface-sensitive techniques, discussed in the following section.

### 4.2 Other Useful Techniques

To provide supporting information to cyclic voltammetry, spectroscopic techniques, including UV–vis,^122^ IR, and Raman,^123−126^ X-ray photoelectron spectroscopy (XPS),^38^ and X-ray absorption spectroscopy (XAS),^127^ are commonly engaged. For example, a triazole formed from azide–alkyne coupling has a diagnostic band in IR spectroscopy, offering a unique feature to confirm the success of the immobilization.^17^ XPS allows for the identification of elements that are present on the surface.^17,96^ UV–vis spectroscopy is also cogent and is often combined with XAS to provide complementary information on the anchored site.^20,65,114^ X-ray absorption near edge structure (XANES) and extended X-ray absorption fine structure analysis (EXAFS) are often employed to provide complementary structural information.^105,114^

Additionally, many techniques allow an *in situ* monitoring of the surface attached species and/or near surface intermediates during a reaction and provide insight into the reaction mechanism, i.e., change in ν(CO) in CO_2_ reduction^80^ or changes in the oxidation state of a catalytically active metal center.^114^ Finally, recent efforts on characterizing interfacial electric fields at electrode/electrolyte surfaces using vibrational sum frequency generation (VSFG) provide an understanding of the electrified interface on a molecular level. A recent comprehensive review emphasized the potential of VSFG in exploring fingerprint bands of surface-adsorbed molecular species compared with other spectroscopic techniques.^118,128^ For example, VSFG was applied to study the strength of the interfacial electric field generated in CO_2_ reduction by a rhenium-functionalized gold electrode.^129^ Investigations on the binding site of the catalyst by sum frequency generation spectroscopy (SFG) and IR spectroscopy provided insight into the strength of the interfacial electric field generated near the charged electrode surface by monitoring the shifts of the carbonyl stretching frequencies and amplitude strongly dependent on the applied potential. The magnitude of the interfacial electric field was calculated to be 10^8^–10^9^ V/m based on experimental data. Remarkably, the order of magnitude of the electric field strength determined in this work resembles the results determined inside active sites in a biological system that bridges the gap between enzymatic, homogeneous, and heterogeneous catalysis.^130,131^

Additionally, surface-enhanced Raman spectroscopy (SERS) represents another powerful tool for *in situ* surface characterization.^132^ To aid the interpretation of the surface-functionalized compound’s performance, comparisons made between the immobilized and the homogeneous analogue using multiple techniques are highly valuable.

## 5 Implications of Surface Immobilization in Electrochemical Catalysis

In Section 3, a brief discussion on the catalytic performance of a constructed hybrid catalytic material was presented based on different immobilization strategies. In this section, we will delve into possible implications after functionalizing the electrode surface with a metal complex to address the sometimes convoluted reaction path and analyze structure–activity relationships. Three aspects to consider for the development and optimization of a molecularly modified electrode in electrocatalysis are presented: (1) the electronic coupling between the electrode surface and immobilized compound; (2) the surface–molecule electrostatic coupling enforced by the local reaction environment; and (3) the molecule–molecule electrostatic interactions engendered by charged substituents on the local structure of the surface attachment. By outlining how the proximate electrode asserts its influence on catalysis, we hope to stimulate critical thinking on the design, development, and optimization of the hybrid construct in real-world applications. Structure–activity correlation is brought up to highlight the possibility of a systematic investigation and manipulation of the hybrid system on a molecular level.

### 5.1 Electronic Coupling between the Electrode Surface and the Immobilized Catalyst

Efficient electron transfer from the electrode to the reactant or redox mediator is a key factor in determining the reaction rate of an electrocatalytic transformation.^9,67,119,121^ A heterogenized molecular catalyst on the electrode decreases the distance for electrons to travel and circumvents the limitation on mass transportation.^9,15^ Understanding the electron transfer pathway helps with the interpretation of the reaction mechanism and ultimately benefits the optimization of the hybrid catalyst. Homogenous electrocatalysts usually undergo an outer-sphere interfacial electron transfer, meaning electrons traverse the double layer between the electrode and redox intermediates dissolved in the solution.^9,21^ Such a process is commonly carried out in two steps: electron transfer and substrate activation. An outer-sphere stepwise electron transfer pathway is also widely adopted by surface-anchored catalysts predominantly depending on the type and length of the linkage. The electrode in this scenario serves as the source or sink of electrons, and the redox behavior of the resulting system is anticipated to be similar to the homogeneous analogue.

**Figure 13:**
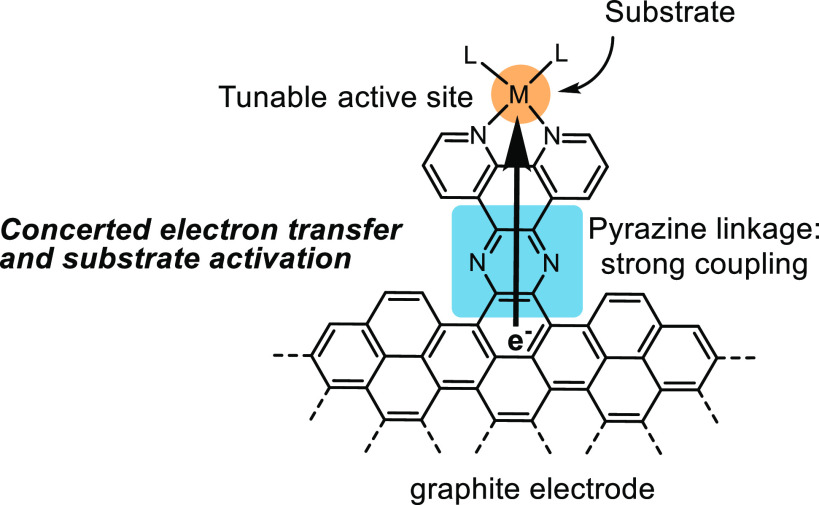
Graphite-conjugated catalyst coupled with the electrode through a pyrazine linkage.^94^ Adapted with permission from ref (94). Copyright 2018 American Chemical Society.

However, when the attached catalyst gets close enough to the surface, for example, through a strongly conjugated linkage (Figure 13), the electronic coupling between the electrode and the surface-attached functionality is strengthened, and as a result, an alteration in the electron transfer pathway may be observed. Inner-sphere electron transfer, commonly observed when a metal electrode is employed in electrocatalysis, becomes available for a strongly electronically coupled system. The molecularly modified electrode directly participates in bond rearrangement under such a scenario without redox mediation.^21^

To gain a deeper insight into the electronic coupling between molecules conjugated to the graphitic surface of a carbon electrode, a series of graphite-conjugated compounds were developed and investigated.^20,21,92−96,114^ Graphite conjugation through amine-quinone condensation formed a conductive pyrazine linkage featuring a strong electronic coupling between the functionalized moieties and the electrode (Figure 13). A fundamental difference in electron transfer was observed for graphite-conjugated catalysts compared to their homogeneous counterparts in electrochemical reactions.^94^ In short, graphite-conjugated catalysts behave more “electrode-like” than “molecule-like”, adapting an inner-sphere electron transfer pathway akin to the metal electrode. Different from homogeneous electrocatalysis, where a redox event is separated from substrate activation, the strong coupling between the electrode and the immobilized metal complex allows for a concerted electron transfer and substrate activation.^93^ In addition, the oxidation state of the transition metal center on the site remains unchanged under electron transfer conditions.

**Figure 14:**
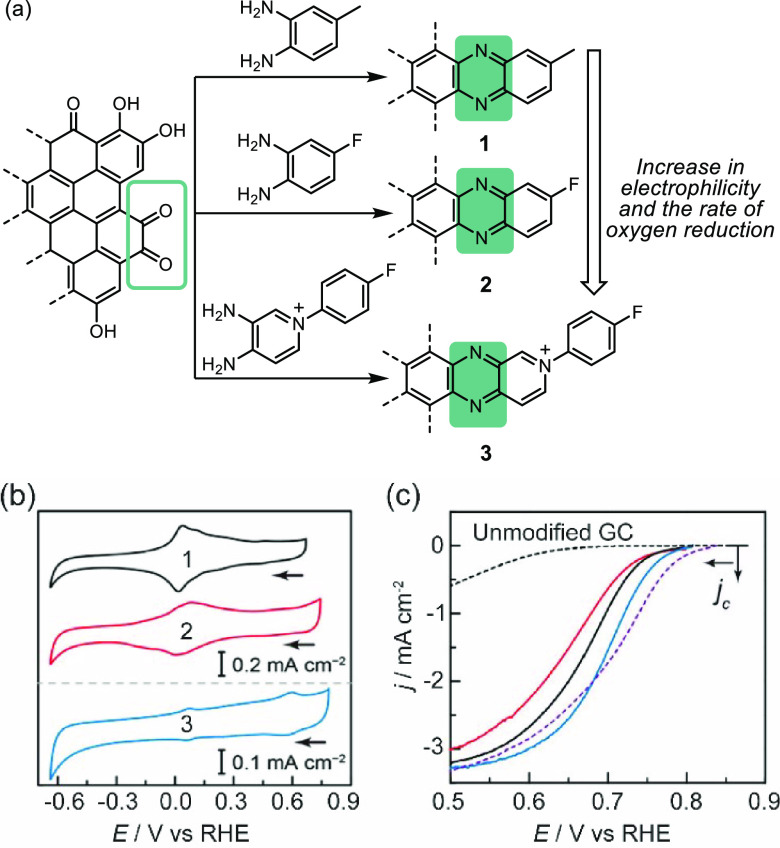
(a) Condensation of *o*-phenylenediamine derivatives with the *o*-quinone edge sites of graphene sheets to generate graphite-conjugated pyrazines. (b) Cyclic voltammograms of **1** (black), **2** (red), and **3** (blue) recorded in an N_2_ saturated 0.1 M KOH electrolyte. (c) Linear sweep voltammograms of **1** (black), **2** (red), **3** (blue), and unmodified GC (dotted black) recorded in an O_2_ saturated 0.1 M KOH electrolyte.^92^ Adapted with permission from ref (92). Copyright 2015 American Chemical Society.

Initial screening of the catalytic activity of the graphite-conjugated catalysts was performed for oxygen reduction reactions. Graphite-conjugated pyrazines obtained from the condensation of *o*-phenylenediamines with *o*-quinones on the edge planes of graphitic carbon electrodes displayed a higher activity in oxygen reduction than the molecular analogues (Figure 14a).^92^ Surface-attached pyrazines showcase reversible redox features in aqueous electrolytes in contrast to untreated electrodes (Figure 14b). Although unmodified glassy carbon electrodes showed activity in oxygen reduction at 0.6 V, graphite-conjugated pyrazines displayed a much higher current at the same voltage, suggesting they were better catalysts under the same conditions despite the fact that the surface site density of electroactive pyrazines, ranging from 14 to 250 pmol cm^–2^, was relatively low (Figure 14c). Most importantly, the rate of oxygen reduction catalyzed by graphite-conjugated pyrazines was found to be positively correlated with the electrophilicity of the pyrazine unit.^21,92^ Changing from the relatively electron-donating methyl substituent to the electron-withdrawing fluoro and aryl-pyridinium substituents of the pyrazine unit resulted in a positive shift in the onset potential and an increase in the turnover frequency, indicating an increasing rate in catalyzing oxygen reduction reactions. This study demonstrated the possibility of modulating the catalytic behavior of a heterogenized system based on the synthetic strategies developed for homogeneous catalysis. Correlations found between the structure of the active sites of the chemically modified electrode and the catalytic performance imply potential molecular control over the active site.

**Figure 15:**
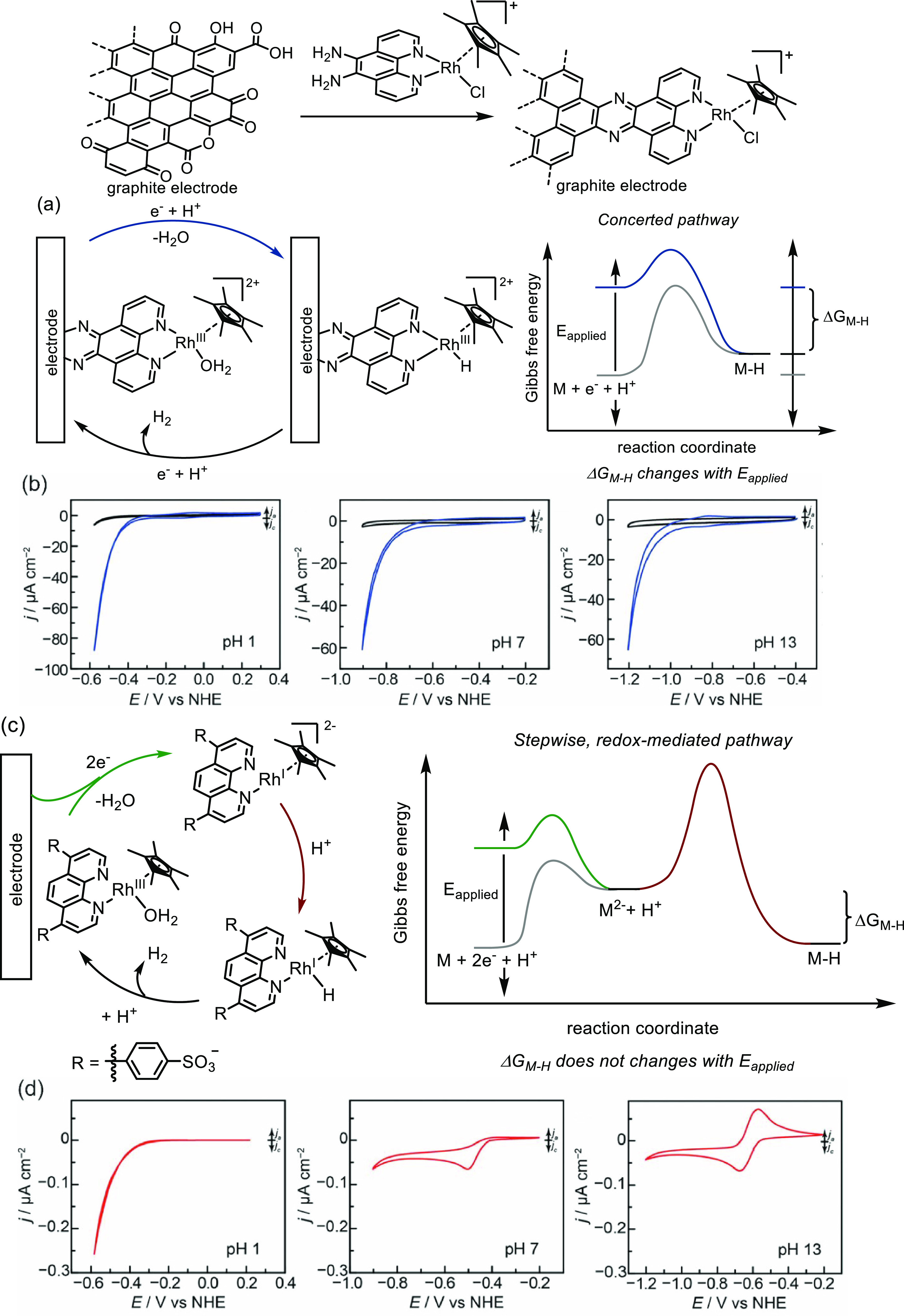
Graphite-conjugated rhodium system. (a) Graphite-conjugated rhodium undergoes concerted electron and proton transfer forming a metal–hydride (M–H) intermediate in hydrogen evolution. (b) pH dependence of hydrogen evolution using GCC-Rh (blue) and GCC-phenazine (black) at pH 1, 7, and 13. (c) Molecular rhodium catalyst undergoes stepwise electron transfer and substrate activation. *E*_applied_ is the applied potential, Δ*G*_M–H_ is the driving force for forming the M–H intermediate. (d) pH dependence of hydrogen evolution using RhCp*(bpds)(OH_2_) at pH 1, 7, and 13 (bdps = bathophenanthrolinedisulfonate).^93^ Adapted with permission from ref (93). Copyright 2019 American Chemical Society.

A closer look into the mechanistic disparity between the stepwise vs concerted electron transfer pathway as a result of the electronic coupling between the electrode surface and the attached metal complex was explored for hydrogen evolution reactions. Figure 15 depicts a graphite-conjugated rhodium complex, which was found to be an active catalyst in an aqueous solution at *all* pH values for hydrogen evolution (Figure 15a,b). Additionally, the catalysis remained rhodium-centered with the oxidation state of rhodium unchanged. In stark contrast, when a molecular rhodium catalyst was employed, the catalysis only operated at low pH values to enable proton transfer after Rh(III) was reduced to Rh(I) (Figure 15c). Experiments further proved that the conjugation to the electrode was essential to maintain the above-mentioned activities. Replacing the ligand backbone of the molecular catalyst with a phenazine moiety was insufficient for catalysis. The experimental observations in hydrogen evolution reactions catalyzed by the graphite-conjugated rhodium catalyst reveal a fundamentally different mechanism compared to the molecular catalyst. As illustrated in Figure 15a, a graphite-conjugated rhodium catalyst (GCC-Rh) undergoes an inner-sphere concerted proton–electron transfer pathway, in which the driving force of forming the Rh–H species is governed by the intrinsic affinity of the rhodium center to the proton. Increasing the applied potential can directly increase the electrostatic attraction of protons to the surface; therefore, the process is not restricted by pH values. However, the molecular rhodium catalyst proceeds *via* an outer-sphere stepwise pathway, where the driving force to form the Rh–H species becomes weaker as the proton donor becomes weaker at higher pH values (Figure 15c). As a result, the molecular rhodium compound can catalyze hydrogen evolution reactions only at low pH values (Figure 15d). Since no redox mediation is needed for GCC-Rh, it could even catalyze hydrogen evolution reactions under conditions where the half potential (*E*_1/2_) of Rh^III/I^ is more positive than the thermodynamic potential for hydrogen evolution. Overall, GCC-Rh operates at extended pH ranges and displays reactivity that is not dependent on *E*_1/2_ (Rh^III/I^) (Figure 15b).

In summary, the graphite-conjugated system enabled a concerted electron transfer and substrate active pathway that is commonly observed for metal electrodes. Meanwhile, a molecular-level control of the active sites can be achieved by modifying the conjugated moiety with molecular precision. However, the condensation of *o*-phenylenediamines with *o*-quinones on the electrode suffers from the low population of surface quinone groups on carbon electrodes as well as applicability toward different types of molecular functionalities.

### 5.2 Electrostatic Coupling of Appended Catalyst to Surface Enforced by Solvent

In the previous examples of graphite-conjugated pyrazines and rhodium complexes, a fully conjugated linkage between the molecular components and the electrode, namely, a conductive phenazine, was indispensable for the strong electronic coupling between the electrode and appended metal complex. However, due to the lack of naturally available *o*-quinones on the electrode surface, the formation of phenazine or pyrazine may be restricted. Under such circumstances, whether a strong electronic coupling between the surface attachment and the electrode through various types of linkage can be achieved regardless of conjugation becomes an intriguing frontier to explore. The use of a solvent in which the molecular moiety is poorly soluble may enforce a concerted mechanism for a weakly conjugated amide-linked cobalt complex.^114^ Chemically modified electrodes derived from a covalent anchoring strategy through an amide linkage were proposed to adopt a stepwise and redox-mediated reaction pathway during electrocatalysis.^120^ The metal centers reach their active states by gaining or losing electrons through an electrostatic potential gradient within the electrical double layer. The electron transfer process is then followed by chemical reactions of the substrates at these active metal sites in the solution. In the comparative study of graphite-conjugated cobalt tetraphenylporphyrin (GCC-CoTPP) and nonconjugated cobalt porphyrin complex (Amide-CoTPP) shown in Figure 9, the superior catalytic activity observed in GCC-CoTPP for oxygen reduction was attributed to the strong electronic coupling conferred by the graphite-conjugated catalyst. However, when a long and flexible aliphatic amide linkage was used, a solvent-dependent concerted electron transfer-substrate activation pathway was observed, similar to the fully conjugated system, but opposite to the nonconjugated system with a short amide linkage.^20,114^

**Figure 16:**
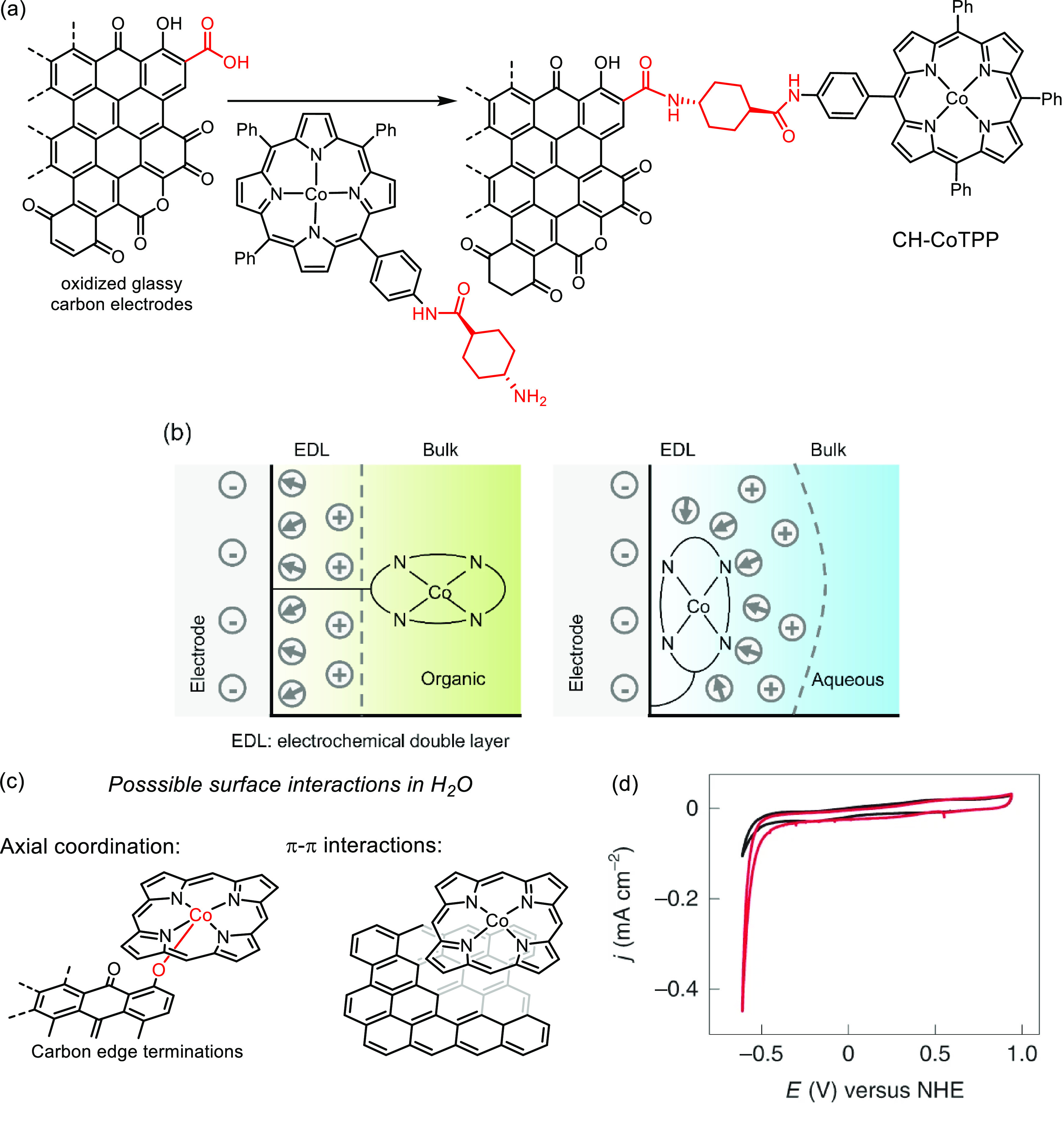
(a) Chemically anchored cobalt porphyrin with an aliphatic tether on the surface of a preoxidized glassy carbon electrode. (b) Interfacial environment of the electrode with chemically anchored cobalt porphyrin in an organic (left) and aqueous (right) electrolyte. (c) Possible noncovalent interactions between cobalt porphyrin and electrode in an aqueous electrolyte. (d) Representation of a cyclic voltammogram of hydrogen evolution using CH-CoTPP (red) in aqueous media (TPP = tetraphenylporphyrin).^114^ Adapted with permission from ref (114). Copyright 2022 Nature Publishing Group.

Experimental studies showed that when a relatively long aliphatic tether was used to anchor a cobalt porphyrin on the surface of a preoxidized glassy carbon electrode, the choice of reaction solvent alters the reaction mechanism (Figure 16). In acetonitrile, an outer-sphere electron transfer corresponding to the Co^II/I^ redox event was observed, consistent with the analogous homogeneous system. As anticipated, it was found to catalyze hydrogen evolution *via* a stepwise pathway, akin to molecular catalysts. In contrast, no cobalt-based redox event was observed by cyclic voltammetry in aqueous media at various pH values. The key difference between the molecularly modified electrode in the two systems is that, in acetonitrile, the homogeneous analogue and the anchored catalyst remained soluble and thus are considered freely solvated and stay outside the double layer. In contrast, the molecular cobalt complex and the anchored compound were insoluble in the aqueous solution and were forced to stay close to the electrode and likely within the double layer (Figure 16b) due to extra surface interactions (axial coordination and π–π interactions, Figure 16c). Investigation of the mechanism of hydrogen evolution reactions catalyzed by surface-attached cobalt tetraphenylporphyrin through a long aliphatic tether (CH-CoTPP) suggested a nonmediated mechanism in aqueous media over a pH range of 0.3–12.8 (Figure 16d). As previously discussed, homogeneous and weakly coupled electrocatalysts that reside outside the double layer undergo a characteristic outer-sphere, stepwise, redox-mediated pathway, in which electrons tunnel through the double layer in response to the potential difference. However, in the case of strong coupling, the energy level of the metal center is manipulated with the electrode in tandem by an externally applied potential. Meanwhile, ions or protons traverse through the double layer in response to a change in the potential. Increasing the applied potential can directly increase the electrostatic attraction of protons to the surface, consequently driving the reaction forward. Nonetheless, the strong coupling between the electrode and the appended catalyst is pivotal to engendering the share of energy levels.

As discussed previously, a strong coupling between the electrode and the surface-attached catalyst can be introduced through fully conjugated chemical bonds (electronic coupling) or preferential surface interactions (electrostatic coupling). The strong electronic coupling described in the graphite-conjugated systems represented by GCC-CoTPP only allows for the concerted pathway by the full conjugation between the surface and appended molecule. To achieve an alternation between different mechanisms, a long and flexible linkage allowing both surface-adsorbed and freely solvated forms of the catalyst is needed. Furthermore, the molecular–surface interaction is profoundly influenced by the solvent. A solvent in which the appended metal complex is insoluble leads to preferential surface interactions, such as axial coordination and π–π interactions, alters the reaction mechanism of the cobalt catalyst immobilized with a flexible linkage from “molecular-like” to “metal-like” causing starkly disparate catalytic activity. Therefore, solvent-induced electrostatic interactions between the anchored molecular catalyst and the electrode surface, as demonstrated in this example, are another important parameter to consider in the design of electrodes modified with molecular active sites.

### 5.3 Ligand Substitution Induced Electrostatic Interactions

The surface-attached cobalt complex with a flexible amide linkage depicted in Figure 16 showcased how the electrostatic interactions between the electrode and the surface-attached metal complex can be enforced by the local environment of the system. Electrostatic interactions are also common among freely dispersed molecules in the electrolyte, especially for molecules bearing charges.^134^ Due to the local electric fields generated by the charged electrode surface, substrates bearing charges tend to diffuse across the solution and accumulate outside the Helmholtz plane.^67^ In heterogeneous CO_2_ reduction reactions, alkali metal cations in the electrolyte, for example, are found near the active site and contribute to the electrostatic stabilization of reaction intermediates. Besides ions in the electrolyte, charged substituents of the appended organometallic complex have the potential to stabilize key intermediates during catalysis as well.^135^ However, only a few investigations on the electrostatic effects induced by the ligand substituents of the surface-immobilized system were reported.^133,135−138^ In this section, we will discuss how ligand substituents could contribute to stabilizing reaction intermediates, in addition to providing electronic inductive effects and reducing catalyst aggregation on the surface.

In homogeneous catalysis, when no external electric field is applied, local electric fields induced by charged groups play an important role in controlling catalytic activity.^139−146^ Additionally, the local structure of the ligand was found to assert its influence on catalysis through electrostatic interactions with substrates and intermediates.^147^ Recent studies on the effect of ligand substituents on molecular electrocatalysis emphasize the importance of considering through-space effects caused by the installed substituents in addition to the inductive effects (through-structure) that are commonly invoked at the molecular level.^138,148^ In other words, the implementation of charged substituents on a molecular functionality being immobilized not only alters the electronic properties of that functionality by donating or withdrawing electrons butalso may induce electrostatic stabilization of key intermediates.

**Figure 17:**
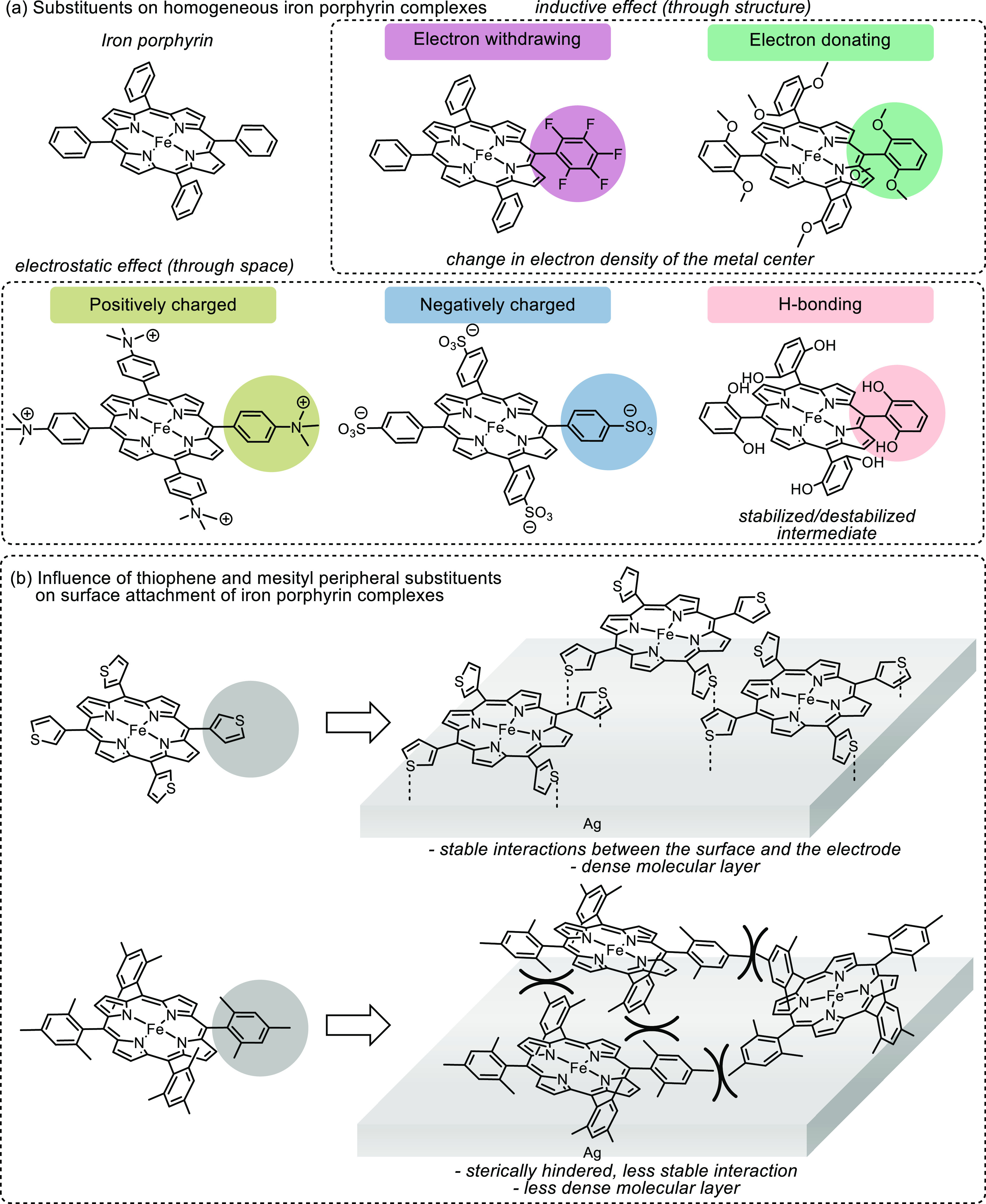
(a) Representations of iron porphyrin complexes with electron-withdrawing, electron-donating, positively, and negatively charged substituents on porphyrin, as well as substituents that possibly introduce H-bonding formation. (b) Homogenous iron complex with thiophenyl created a relatively dense molecular layer on an electrode surface, whereas bulky mesityl substituents resulted in a less stable interaction with the electrode surface and created less dense surface coverage.^133^ Adapted with permission from ref (133). Copyright 2019 American Chemical Society.

The major challenge in interpreting the implications of ligand substitutions remains in disentangling inductive (through-structure) and electrostatic (through-space) effects.^135,146,148−150^ A series of homogeneous substituted iron porphyrins was synthesized to investigate inductive and electrostatic effects in CO_2_ reduction. Installing an electron-withdrawing substituent was found to lower the overpotential by reducing the electron density around the metal center. In contrast, electron-donating substituents were observed to behave the opposite.^148,149,151^ A linear correlation between log(turnover frequency) and the standard potential was found when the electronic inductive effect of the substituents was taken into account. However, incorporating charged substituents was found to stabilize key intermediates through electrostatic attraction or repulsion, causing a significant deviation of those catalysts from the linear correlation.^148^ In other words, the presence of electron-donating and electron-withdrawing substituents in iron porphyrin complexes resulted in well-understood inductive effects, whereas the presence of positively and negatively charged substituents on the ligand resulted in significant through-space electrostatic effects (Figure 17a).^133,135,149^ Additionally, the presence of hydrogen bonding between the ligand and substrate could also help stabilize intermediates. Similar findings were observed in a substituted cobalt-porphyrin, where distinct trends of log(turnover frequency) and Hammett constants (σ) were found for neutral and cationic substituents.^138^ Neutral substituents, including both electron-withdrawing and electron-donating substituents, were found to affect inductively the electronic properties of the cobalt complex, while the cationic substituents deviated, indicating additional effects. Cationic substituents were again postulated to provide an electrostatic stabilization of the intermediate in the rate-determining step.

When attaching a substituted homogeneous metal complex on an electrode surface, the effects of sterically hindered substituents on the loading density and aggregation of the catalyst have to be considered.^133,137^ Gotz et al. presented a study on the influence of mesityl and thiophene peripheral substituents on surface-appended iron porphyrin complexes (Figure 17b).^133^ It was found that iron porphyrin complexes with thiophenyl substituents created a dense and planar molecular layer on a silver electrode, resulting in a facile electron transfer *via* Ag–S covalent bonds. On the contrary, bulky mesityl substituents caused less dense coverage of the molecular layer and created less stable interactions. It is important to note that the bulky mesityl groups, which were proven to prevent catalyst aggregation and improve catalytic behavior in solution, exerted a negative effect after immobilization.

**Figure 18:**
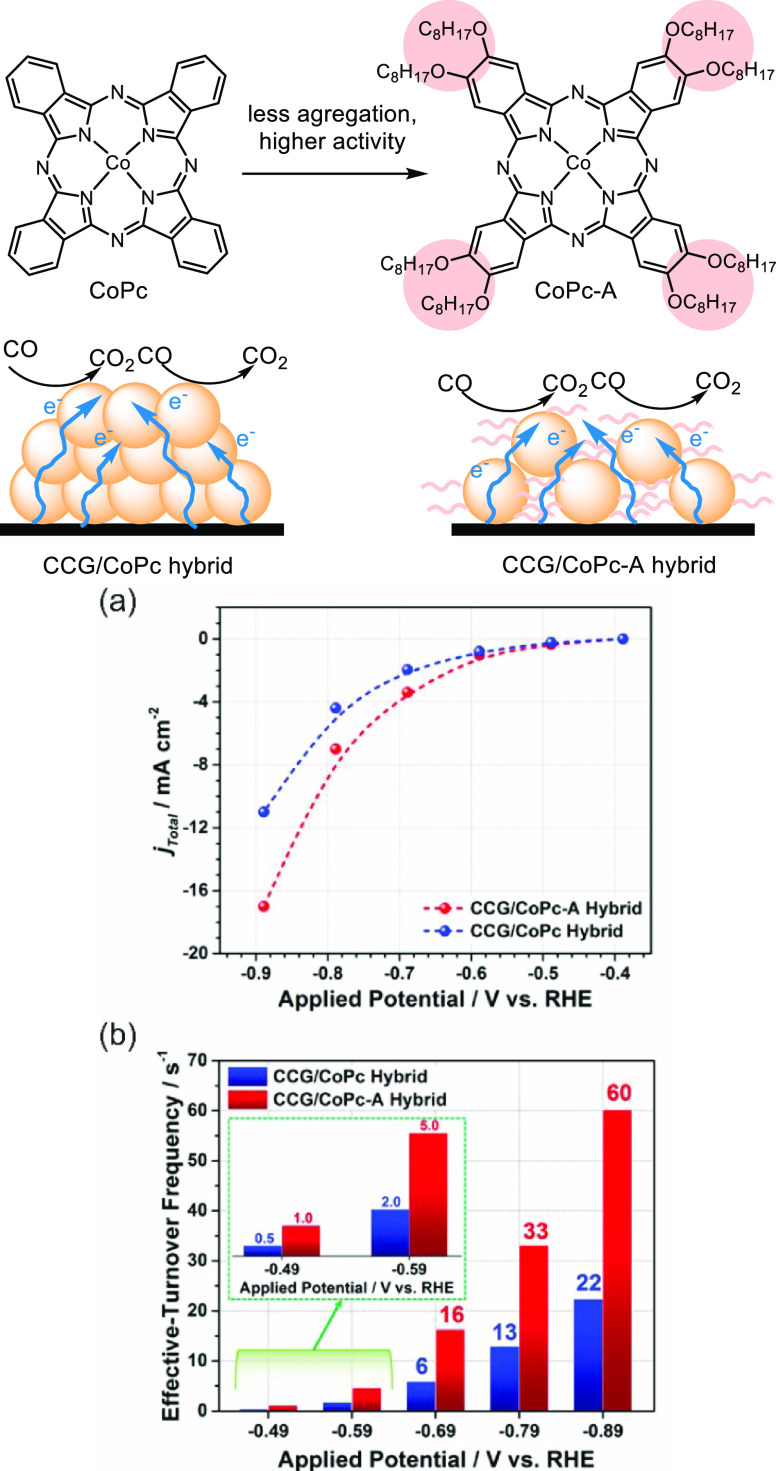
Ligand substitution of an immobilized cobalt complex reduces aggregation; CCG stands for chemically converted graphene. (a) Total current density for CCG/CoPc-A and CCG/CoPc hybrids in a CO_2_ saturated electrolyte. (b) Effective TOFs for CO production by CCG/CoPc (blue bar) and CCG/CoPc-A (red bar) hybrids at different applied potentials.^137^ Adapted with permission from ref (137). Copyright 2019 American Chemical Society.

However, introducing sterically hindered substituents on the ligand was found to suppress catalyst aggregation and increase catalytic activity in the case of an immobilized cobalt complex, despite the decrease in the coverage of the catalyst. A prolonged catalytic lifetime was observed for the immobilized cobalt octaalkoxyphthalocyanine (CoPc-A) catalyzed CO_2_ reduction (Figure 18).^137^ In addition, a higher total current density was observed for electrochemical CO_2_ reduction with chemically converted graphene derived from cobalt octaalkoxyphthalocyanine (CCG/CoPc-A) than for the less sterically hindered Co(II) phthalocyanine (CCG/CoPc) under the same conditions (Figure 18a). In agreement with this observation, higher TOFs of CO production were found for CCG/CoPc-A hybrid than CCG/CoPc (Figure 18b).

In this section, we discussed important aspects of investigating the electronic and electrostatic coupling between the electrode surface and the attached catalyst to maximize the combined advantages of the heterogeneous support and homogeneous metal complex. A comprehensive understanding of electron communication and other possible static interactions within the system helps the design, development, and optimization of functionalized electrode materials. By presenting the inductive, steric, and static effects of ligand substituents, we emphasized that the local structure of the immobilized complex, along with the local environment of the system, is an effective handle to modulate catalytic activity. Although efforts are still needed to interpret the effect of ligand substituents beyond electronic induction, correlating the structure of the attached entity to its catalytic activity introduces the possibility of precise manipulation of the catalytic active site on the modified electrode surface.

## 6 Applications beyond Energy Conversion

In the previous section, we focused on presenting potential applications of molecularly modified electrodes in electrocatalysis concentrating on redox reactions related to energy conversions with a brief discussion on possible electrostatic interaction between the surface and the attached molecule. In this section, we will primarily focus on the applications beyond energy conversion. In addition to current-initiated electrocatalytic processes, externally applied electric fields may also facilitate catalytic transformations mimicking the powerful electric fields in enzyme catalysis.^131^ Catalysis enabled by an externally applied electric potential has just started to emerge as a viable method in the synthetic chemistry toolbox.^129,152−155^ Furthermore, a patterned electrode provides a platform to achieve spatiotemporal control over catalytic processes by changing the current and the voltage.^40,132^

### 6.1 Electrostatic Catalysis Enabled by Applying an Electric Potential

An external electric field refers to the uniform electric field generated by a voltage bias.^134^ Aligning an electric field along a specific bond axis can either elongate or shorten the bond.^156^ By precisely controlling the orientation of an external electric field along a reaction axis (the direction of bond breaking and bond-forming), a reaction can be accelerated or slowed down.^134,156^ In nature, many enzymatic reactions rely on a preoriented local electric field for electrostatic catalysis.^131^ Efforts have been made to mimic the electrostatic interactions in enzymes and apply them to synthetic chemistry. Detailed discussions on implementing external electric fields, oriented external electric fields, and electrostatic interactions in wide-ranging applications have been included in recent reviews.^134,157−161^ However, common strategies employing scanning tunneling microscopy (STM) to harness oriented external electric fields in catalysis, for example, suffer from scalability. As an alternative, a design of electric cells with “parallel plates” has been developed to demonstrate the possible application of electrostatic field in catalysis.

**Figure 19:**
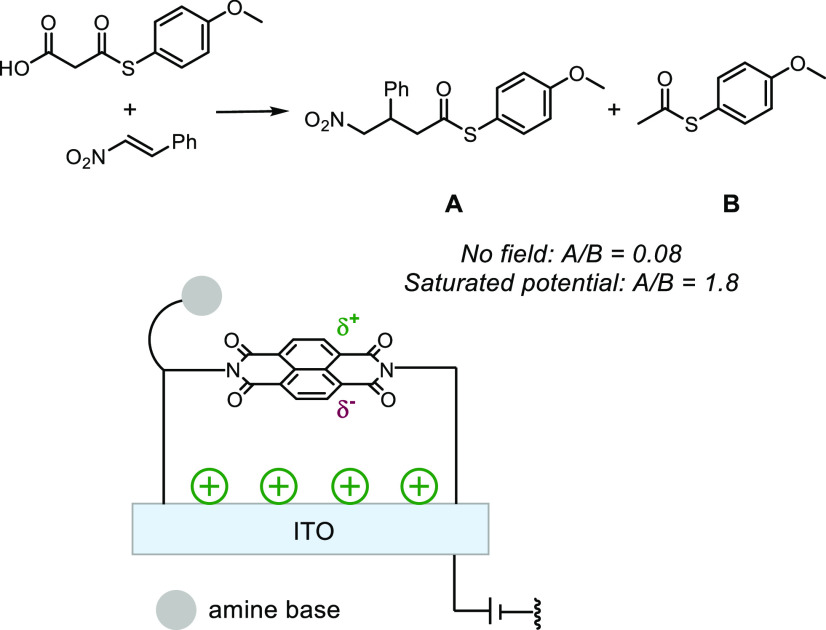
Anion-π catalysis assisted by an electric field.^162^ Adapted with permission from ref (162). Copyright 2017 American Chemical Society.

In non-Faradaic electrochemical cells (no current), interfacial electric fields exist near the surface of charged electrodes where ions gather around and create a double layer in response to an electric field.^134^ Recently, naphthalenediimide was functionalized on a conductive ITO surface to expand the range of electric field-assisted catalysis. The anion-π catalysis of the addition of a malonic acid half thioester to enolates was explored (Figure 19).^162^ Applying a positive potential resulted in the shift in product selectivity toward the unfavored product **A**, compared to when no potential was applied. It was proposed that the externally applied electric field polarized the appended catalyst to increase the recognition of the reaction intermediate that leads to the formation of **A**.^163,164^ Therefore, an inversion of selectivity toward the unfavored product **A** was observed.

**Figure 20:**
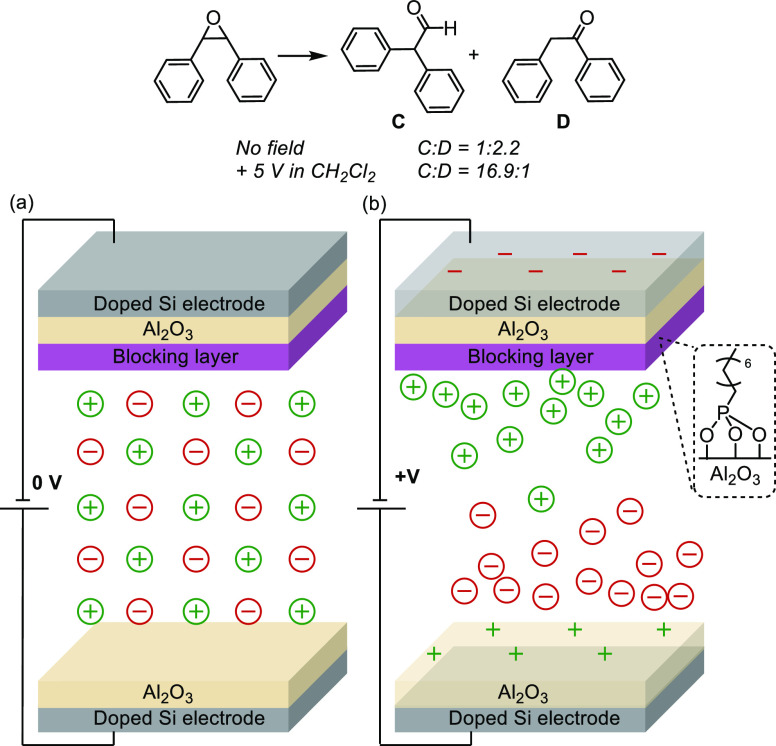
Selectivity in epoxide rearrangement achieved by an externally applied electric field in a parallel plate cell.^165^ Illustration of the metal oxide–electrolyte interface at 0 V (a) and positive voltage (b) showing a significant voltage-dependent electric field at the interface. At 0 V, ions are assumed to distribute evenly at the interface, whereas, in the presence of an applied voltage, ions move toward the polarized plates generating an interfacial electric field as a result. Catalysis was confined to the bottom Al_2_O_3_ layer by blocking the top layer with a monolayer of alkylphophonic acid. Adapted with permission from ref (165). Copyright 2012 American Chemical Society.

The study of naphthalenediimide functionalized ITO surfaces in electric-field-assisted catalysis demonstrates the potential of altering selectivity by inducing field–dipole interactions. However, applying large electric fields to reactions and studying the corresponding effects remains challenging in many cases due to the lack of appropriate experimental setups.^165^ As a consequence, a parallel plate cell that allows the application of a large electric field to the system was developed. Adapting this setup, an electric field-induced change in selectivity in an Al_2_O_3_-catalyzed epoxide rearrangement was observed (Figure 20).^165^ A thin layer of Al_2_O_3_ on a p-doped Si electrode in combination with a counter electrode of alkylphosphonic acid/Al_2_O_3_/Si resulted in a parallel plate cell that allows the investigation of the effects of the electric field without a flow of current. In the absence of an applied electric field, charges are assumed to distribute evenly at the metal oxide–electrolyte interface (Figure 20a), whereas when a voltage is applied, plate surfaces are polarized engendering an electric field at the interface (Figure 20b). This setup can be used to investigate electric field-assisted nonredox reactions since there is no direct supply of electrons generated by the cell.

The change in product ratio of *cis*-stilbene oxide rearrangement catalyzed by Al_2_O_3_ under an applied potential in the designed parallel plate cell was examined. When no potential was applied, the reaction led to a **C**/**D** ratio of 1:2.2. An increasing ratio of aldehyde to ketone was observed when the applied potential was less than −3 V or more than 3 V compared to no potential applied. Such change in the product ratio could be reinforced by selecting the best solvent: in CH_2_Cl_2_, a **C**/**D** ratio of 16.9:1 was observed indicating a 63-fold enhancement compared to the result obtained in CH_3_CN. The greater selectivity observed in CH_2_Cl_2_ was attributed to a compact electrochemical double layer formed in CH_2_Cl_2_, causing larger electric fields at the oxide surface.^119^ The use of an insulating coating decouples the electrostatic effects from the electrochemical effects allowing the analysis of electric field-induced catalysis without the interference of current. Thus, the change in selectivity solely resulted from the change in electric potential, showing the irreplaceable role of the interfacial electric field.

**Figure 21:**
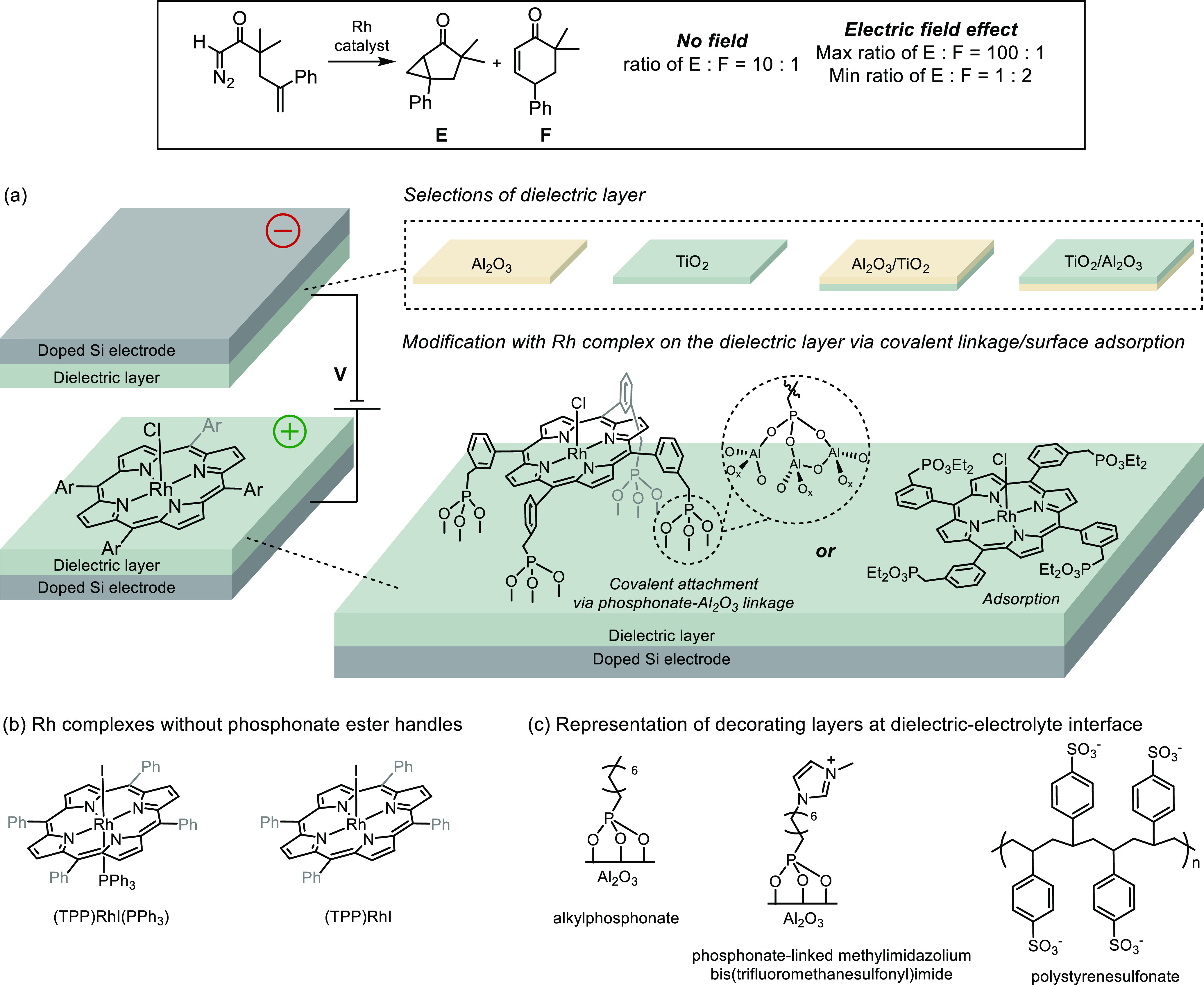
Change in product ratio induced by an electric field for rhodium-catalyzed cyclopropanation reaction. (a) Schematic representation of rhodium complex immobilized between parallel plates ranging from Al_2_O_3_, TiO_2_, Al_2_O_3_/TiO_2_, and TiO_2_/ Al_2_O_3_*via* covalent attachment or surface adsorption. (b) Rhodium complexes without phosphonate ester handles can only be localized *via* surface adsorption. (c) Decorating layers at the dielectric–electrolyte interface either block the interactions between Rh and surface or increase the ionic strength of the interface.^64^ Adapted with permission from ref (64). Copyright 2013 American Chemical Society.

Taking advantage of the ingenious design of the parallel plate cell, carbene rearrangement reactions catalyzed by adsorbed and immobilized rhodium catalysts were studied in the presence of interfacial electric fields (Figure 21).^64^ Silicon electrodes coated with metal oxide layers were used as the opposing plates with a rhodium porphyrin catalyst in between the dielectric–electrolyte interface. The phosphonate ester-modified rhodium complex can be covalently attached *via* a phosphonate-oxide bond, or physically adsorbed on the oxide surface. Applying voltage to the parallel plate cell resulted in changes between the cyclopropanation product **E** and the insertion product **F** (Figure 21).

In the absence of an applied voltage, the Rh-catalyzed intramolecular carbene reaction from diazoketone formed products **E** and **F** in an approximate ratio of 10:1. Upon applying a voltage, the covalently attached Rh catalyst induced an *increase* in the ratio up to >100:1 at +4 V as a result of increasing the charge density when TiO_2_ was used, indicating an almost absolute selectivity toward **C**. On the contrary, the rhodium functionalized Al_2_O_3_ surface caused a *decrease* in the ratio of **E**/**F** regardless of the direction of the potential applied. Furthermore, the effect of the electrical and chemical properties of the dielectric layer was investigated using different compositions of the plate: pure Al_2_O_3_ and TiO_2_ on top of the silica, 10 Å of TiO_2_ atop 40 Å of Al_2_O_3_ (“TiO_2_/Al_2_O_3_/Si”), and 10 Å of Al_2_O_3_ atop 40 Å of TiO_2_ (“Al_2_O_3_/TiO_2_/Si”) (Figure 21a). The rhodium complex attached to Al_2_O_3_ and Al_2_O_3_/TiO_2_/Si caused a *decrease* in the product ratio, whereas TiO_2_ and TiO_2_/Al_2_O_3_/Si resulted in an *increase* in the ratio. These findings emphasize the importance of examining different surfaces for reaction optimization. In this case, the oxide surface determines the direction of the potential-dependent selectivity change.

A comparison between covalently attached versus surface adsorbed Rh catalysts was conducted with a similar trend in selectivity change observed: in most cases, both covalently immobilized and physically absorbed Rh complexes on Al_2_O_3_ coated surfaces caused a *decrease* in the ratio of **E**/**F**, whereas on TiO_2_ surfaces, an *increase* in the ratio was found (Figure 21a). However, when a rhodium complex with triphenylphosphine ((TPP)RhI(PPh_3_), Figure 21b) was used, a *decrease* in the ratio was observed for *both* Al_2_O_3_ and TiO_2_. A similar observation was found when a blocking layer of alkylphosphonate (see Figure 21c for a representation) was deposited onto the surface, interrupting the interactions between the surface and the rhodium center. Lastly, increasing the ionic strength of the interfacial region by codepositing the rhodium complex with a decorating layer of phosphonate-linked methylimidazolium bis(trifluoromethanesulfonyl)imide with or without a layer of polystyrenesulfonate resulted in significant changes in magnitude in product ratio.

This study demonstrates an incredible control of otherwise challenging selectivity of a Rh-catalyzed intramolecular carbene reaction, achieving the following:

1. selectivity dependent on the dielectric properties of the surface, as observed from the difference in Al_2_O_3_ versus TiO_2_
2. similar selectivity with covalently attached and surface-adsorbed rhodium complexes with or without a phosphate ester handle. However, the presence of an extra phosphine ligand or a layer of phosphonate can block the necessary interactions between the surface and the metal center leading to the inversion of activity. For instance, an *increase* in the ratio of **E**/**F** was observed when (TPP)RhI on TiO_2_ was used, whereas a *decrease* was found when (TPP)RhI(PPh_3_) on TiO_2_ was employed.
3. selectivity by increasing the ionic strength of the surface by codepositing decorating layers of phosphonate-linked methylimidazolium bis(trifluoromethanesulfonyl)imide and/or polystyrenesulfonate compared to metal oxide surfaces without those decorating layers

### 6.2 Alternating Electronic Properties of Surface Attached Substrates by an Incrementally Applied Voltage

A molecularly modified electrode has the potential to play dual roles in electrochemical processes: providing ready access to electrons and/or enforcing a catalytic process by an external/local electric field. Being able to adjust the applied potential over time creates an opportunity to achieve temporal control in catalysis.^40,166,167^ However, it is undeniable that most applications of molecularly modified electrode catalysts are limited to the energy sector, and breakthroughs are rarely reported in other areas.^6^

**Figure 22:**
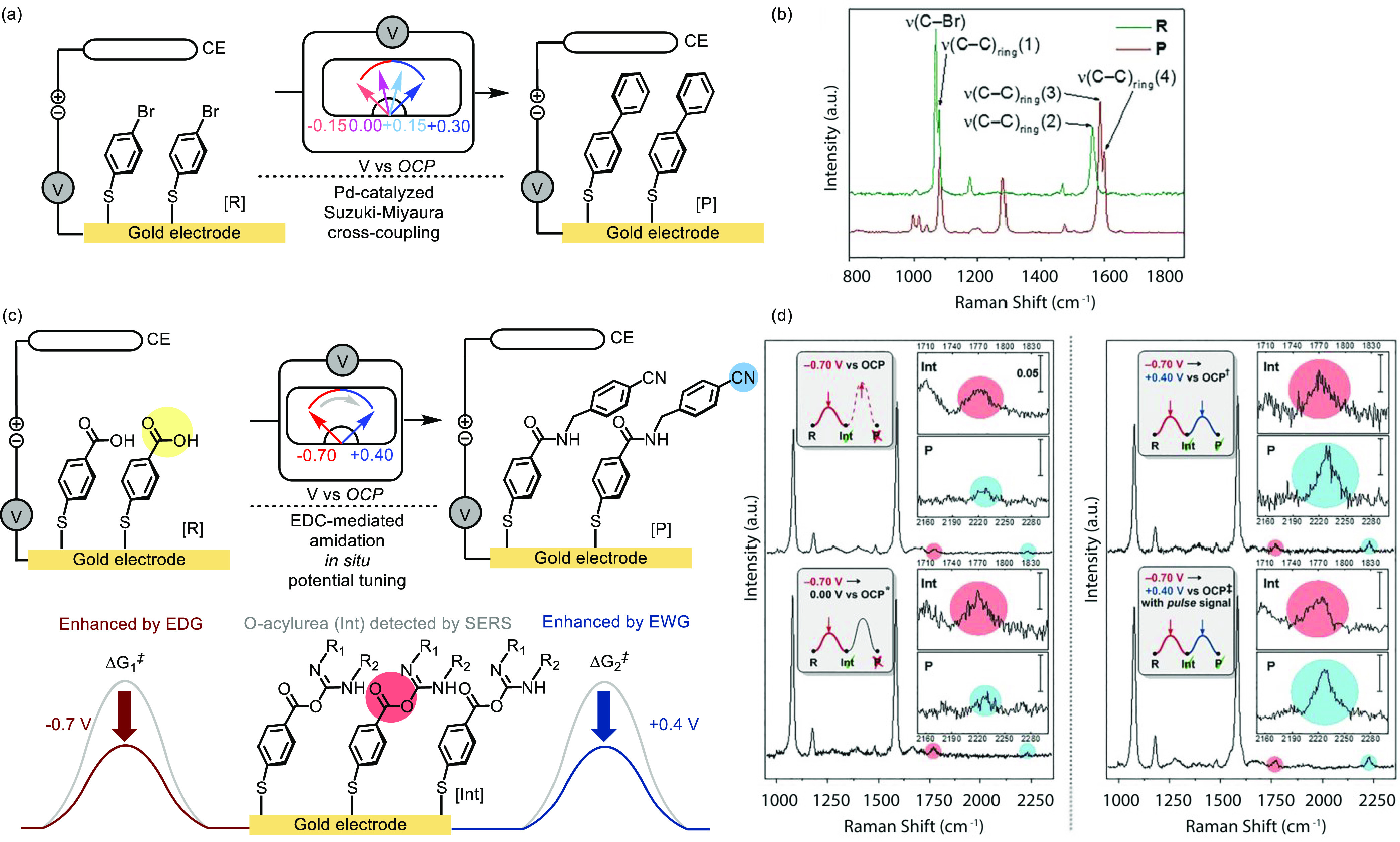
(a) Palladium-catalyzed Suzuki–Miyaura cross-coupling reaction, and (b) SERS of the reactant, 4-bromobenzenethiol (green line, R), and product, biphenyl-4-thiol (brown line, P). (c) Carbodiimide-mediated amidation reaction (CE, counter electrode; EDG, electron-donating group; EWG, electron-withdrawing group; OCP, open circuit potential; [R], reactant; [P], product; Int, intermediate; Δ*G*^‡^, Gibbs free-energy barrier). (d) Left: Representative SERS of EDC-mediated amidation with −0.70 V (top graph) and a sequence of −0.70 V to OCP (bottom graph). Right: Representative SERS of EDC-mediated amidation after a sequence of −0.70–+0.40 V (top graph) and pulsed potential condition (bottom graph) (EDC = 1-ethyl3-(3-(dimethylamino)propyl)carbodiimide).^132^ Adapted with permission from ref (132). Copyright 2020 American Association for the Advancement of Science.

The rate of a catalytic reaction affected by the electronic properties of the molecular components in the reaction system through inductive effects has been modulated by the prominent and pervasive use of functional groups featuring electron-donating/withdrawing characteristics.^168^ However, each substrate/catalyst bearing a unique functional group imposing a specific inductive effect requires laboratory synthesis and may not be easily accessible. Computational studies of functional group derivatives can assist but still require experimental validations. Due to the limitations on the number of experimentally accessible functional groups and their associated electronic properties, a discontinuity in the inductive effects is inevitable and cannot be alleviated using the traditional organic synthesis approach. In a typical electrochemistry setup, however, the voltage applied can be tuned incrementally and continuously. An unprecedented example took advantage of the electric potential to replace the functional groups that impose different inductive effects. The electronic properties of gold-bound substrates were manipulated by applying different potentials. The modified surface was then investigated in cross-coupling and amidation reactions (Figure 22).^132^ Without using electron-donating or withdrawing substituents, the electronic properties of the molecules being immobilized on the gold electrode could be tuned by applying different voltages: applying a negative potential imitated electron-donating groups, whereas a positive potential imitated electron-withdrawing groups. For example, in a Pd-catalyzed Suzuki–Miyaura cross-coupling reaction (Figure 22a), the reaction rate was slowest at +0.3 V and then increased upon applying a negative potential. The highest activity was reached at −0.15 V, as monitored by surface-enhanced Raman spectroscopy (SERS). SERS of the reactant and product were depicted in Figure 22b. It was observed that applying a negative voltage to mimic an electron-donating group can enhance the rate of the Suzuki–Miyaura reaction.

Continuous and *in situ* tuning of the electronic property of the substrate over time poses a significant challenge to common synthetic approaches. However, modifying the substrate’s electronic properties during a catalytic cycle as discussed above showed promising results, particularly in enhancing the activity for the steps that require opposite electron inductive effects. As illustrated in Figure 22c, an amidation reaction is accelerated when both the nucleophilicity of the carboxylic acid oxygen and the electrophilicity of the carbonyl carbon in *o*-acylurea are enhanced. Therefore, applying a negative voltage first and then inverting to a positive voltage should facilitate the overall reaction. Consequently, a −0.7 V potential that was switched to +0.4 V midway through the reaction did increase indeed the peak intensity of the desired product in SERS (Figure 22d). This study showcases the possibility of utilizing electrodes as functional groups of the immobilized molecules by switching the voltage between positive and negative values to mimic electron withdrawing and donating effects. We envisage that such an approach may facilitate the deduction of structure/property-activity correlation for a designated system. It is important to highlight the indispensable role of SERS in this study as a tool for both *in situ* monitoring of the reaction in addition to the capability of characterizing the material. Through its capability to perform *in situ* measurements, SERS facilitates the discovery of new chemistry on the functionalized surface and provides a deeper understanding of the reaction mechanism.

### 6.3 Redox-Switchable Polymerization Mediated by an Iron Complex Immobilized on an Electrode Surface

Within the context of efficient and sustainable transformations, catalyst engineering strategies are evaluated based on atom economy, step economy, and redox economy.^169^ Replacing chemical oxidants and reductants is one of the major driving forces to implement electrocatalysts in chemical production. Immobilization of a homogeneous electrocatalyst surmounts the barrier of separating and recycling the catalyst from the products. In this context, a chemically functionalized electrode, coupling the efficient electron transfer from the electrode and the molecularly modifiable catalytic active sites of a metal complex, for instance, features a promising catalytic material.

Electrochemical triggers (current/potential) have been recognized as powerful external stimuli to control multistep transformations.^170−173^ Inspired by Nature, where a series of reactions occur in parallel with minimal undesired interference among each other, scientists constructed variants of multicatalytic systems encompassing stimuli-responsive catalysts to ensure the necessary spatial and temporal control over multiple processes being conducted in tandem.^174−180^ In particular, products with higher value and complexity such as polymers can be readily synthesized from simple building blocks in a controlled manner.^169,171−173,181^ Light, mechanical forces, redox, and electrochemical switches embedded in the catalyst design confer an on/off-type regulatory function.^171,178^ Among those, the precise control over an applied potential as an electrochemical switch represents a promising approach toward temporal control.^182^

**Figure 23:**
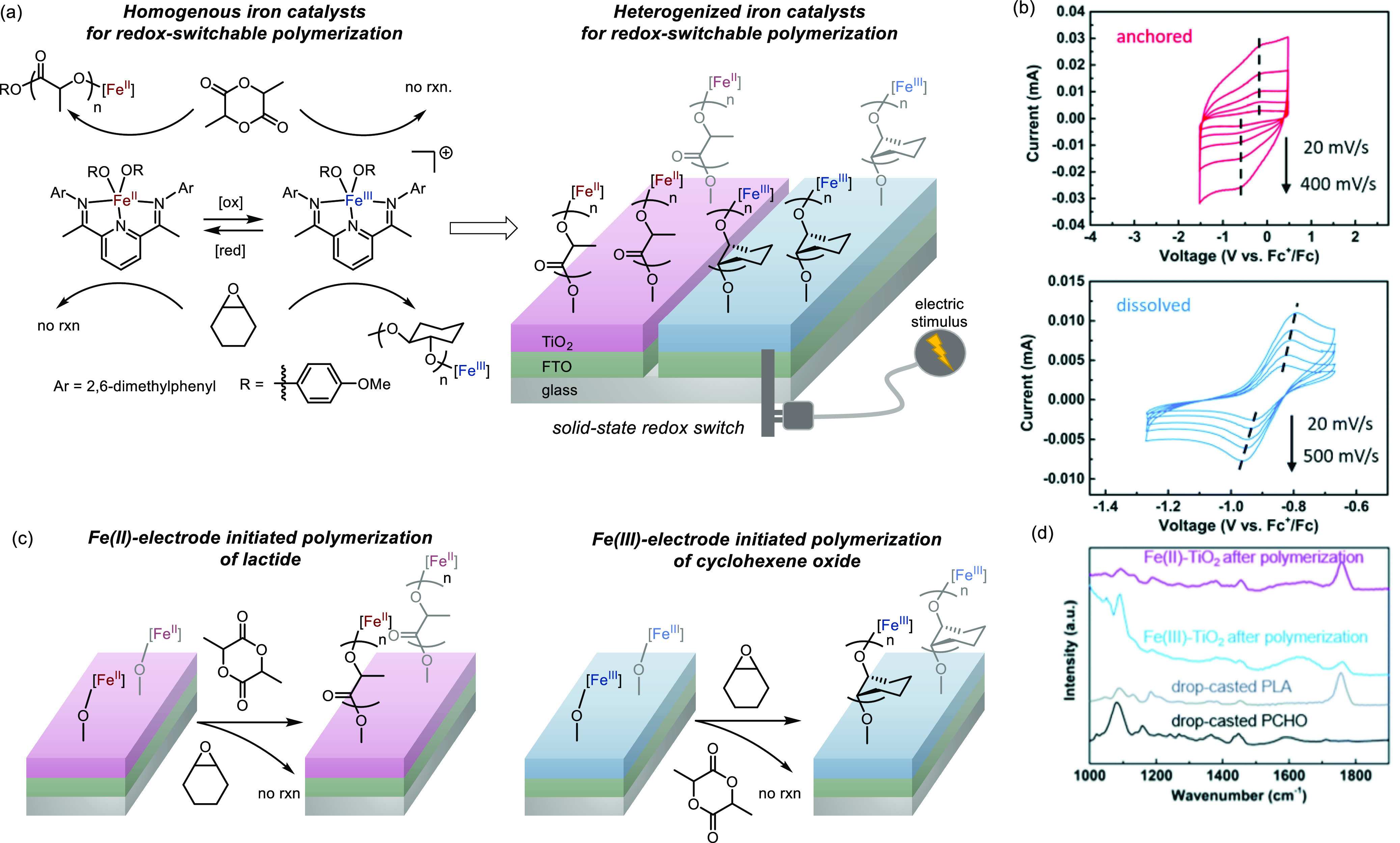
(a) Iron-based catalysts for redox-switchable polymerization of lactide and cyclohexene oxide, homogeneous and heterogenized on an electrode surface. (b) Cyclic voltammetry of an Fe(II)–TiO_2_–Ti mesh electrode (top) and a molecular bis(imino)pyridine iron bisphenoxide complex in solution (bottom) with varying scan rates. (c) Surface-initiated poly(lactide) from Fe(II)-modified TiO_2_ electrode (left) and poly(cyclohexene oxide) obtained by oxidizing the surface Fe(II) complexes Fe(III) (right). (d) FTIR spectra of the functionalized surface after polymerization confirming the formation of surface-initiated polymers in comparison with drop-casted polymers on the surface.^40^ Adapted with permission from ref (40). Copyright 2021 The Royal Society of Chemistry.

Electrochemically control of molecular catalysts in switchable polymerization has been reported for atomic transfer radical polymerization (ATRP),^183^ cationic polymerizations,^184^ and ring-opening polymerizations.^166,167,185,186^ The selective application of an electric stimulus to trigger redox events at a redox-active metal complex provides an alternative way to chemically controlled redox events. In particular, electrochemical control in redox-switchable polymerization reactions alleviates the use of a stoichiometric amount of redox reagents in these processes. It further enables multiple switches by discrete manipulation of the applied potential. Switching between reductive and oxidative potentials allows for on/off control of the polymerization process, control over molecular weights and dispersity, as well as over the stereochemistry and composition of the polymer chain.^40,166,167,183−187^ However, homogeneous catalysts involved in electrochemically controlled switchable polymerizations are limited by mass transport since the catalysts have to diffuse closer to the electrode surface to be activated. Localizing the catalyst near the electrode surface could circumvent this issue. In that context, the combination of electrode supports and molecular metal complexes may address both spatial and temporal control in polymerization reactions. An electrochemically controlled surface-initiated polymerization was possible by using a bis(imino)pyridine iron(II) complex anchored onto titanium oxide nanoparticles (Figure 23a).^40^ The surface-supported redox-active iron complex was achieved by depositing the functionalized titanium oxide nanoparticles onto a fluorine-doped tin oxide surface, serving as the electrically addressable surface for electrochemical polymerization. The functionalized electrode surface was characterized by various methods. Particularly, cyclic voltammetry revealed both oxidative and reductive peaks of the surface-attached iron complex with a 0.5 V positive shift in the half-wave potential relative to the molecular iron complex due to the less electron-donating capability of the metal oxide surface (Figure 23b). The reactivity of the molecular iron complex toward ring opening polymerization of lactide (LA) and cyclohexene oxide (CHO) was maintained after surface functionalization. Both homogeneous and heterogenized iron(II) complexes react with lactide in the reduced state. Oxidizing the iron center to iron(III) by using a chemical oxidant resulted in no activity toward lactide but initiated the polymerization of CHO (Figure 23c). The surface-initiated polymerization was confirmed by Fourier-transform infrared (FTIR) spectroscopy that showed the characteristic bands of polymers (C=O stretching at around 1750 cm^–1^ for PLA and strong C—O stretching at around 1095 cm^–1^ for PCHO) grown from the surface that were comparable to those of drop-casted polymers on the surface (Figure 23d). Applying an oxidative potential to replace chemical oxidants resulted in the same switchable behavior for both homogeneous and surface-anchored iron complexes. A cyclic voltammetry study of the heterogenized iron complex suggested redox events associated with surface-bound species similar to the homogeneous case. Additionally, the polymer chains remained attached to the surface.

Inspired by the selective behavior of electrochemically controlled iron(II)/(III) catalyzed polymerization, a substrate containing two electrically isolated domains was used to demonstrate the spatial control of polymer composition. Exposing one domain to an oxidizing potential while the other domain remained untouched resulted in an iron(III) and iron(II) cofunctionalized electrode surface, where the chemoselectivity toward different monomers remained undisrupted: the growth of polylactide was observed on the untouched domain containing iron(II), while polycyclohexene oxide was obtained on the oxidized domain of iron(III). Overall, a spatially controlled pattern of polymers was achieved on the electrically defined surface.

This study demonstrates a successful case of electrochemically controlled polymerization *via* a molecular functionalized electrode surface in which an electrical potential altered the redox state of the immobilized catalyst, affecting the catalyst’s reactivity and enabling a selective polymerization of two monomers. As a result, a spatially controlled surface polymerization pattern was achieved by switching the oxidation state of the iron complexes on two isolated domains in one system.

## 7 Summary and Outlook

Throughout this review, we tried to convey the advantages of using a functionalized electrode as a hybrid material for electrochemical catalysis. We started by demonstrating strategies for surface immobilization of molecular catalysts categorized by the type of electrode support and viable linkages based on anchoring handles. We discussed several commonly employed conductive materials such as metal and metal oxides. We highlighted carbon-based electrodes due to their conductivity, stability, and availability. Common strategies being applied to modify each type of electrode material were surveyed, along with techniques for characterizing the material after fabrication.

To accommodate the available anchor sites on the electrode surface, complementary modifications of molecular components with necessary functional groups are required. The selection of an appropriate immobilization method depends on the type of reactions and interactions that may occur between the surface and the molecular functionality to be appended. The resulting linkage and/or molecular–surface interaction plays an important role in manipulating electron transfer and determining the reaction mechanism. With an overview of some recent reports on electrochemical transformations related to energy conversion, we compared the effect on the catalytic activity of different linkages/interactions between the anchored metal complex and the electrode. Modifications at the molecular level may result in inductive electronic effects, steric effects, as well as electrostatic effects. Overall effects on the catalytic activity of a hybrid system need to be evaluated in comparison to the homogeneous analogue to get a full picture of the newly established system.

In Sections 5 and 6, we discussed examples of employing functionalized electrodes in gas–liquid electrocatalysis (oxygen reduction, CO_2_ reduction, hydrogen evolution, etc.), potential applications in electric field assisted catalysis, *in situ* modification of electronic properties of the surface attachment, and spatiotemporally controlled electrode surface-initiated polymerizations along with showcasing the unique advantages of using a hybrid catalytic material. Although the primary field of application for functionalized electrodes remains to be reactions related to energy conversion, molecularly modified electrodes have a great potential in the electrocatalytic valorization of organic molecules^188^ and electrochemical synthesis beyond the scope of this review.^3,159,189−198^

We hope to encourage further discussion on combining the merits of molecular catalysts and electrode supports to achieve the optimal activity, selectivity, and durability for a wide scope of chemical transformations. To provide a reference point for beginners in the field, we summarize the following learning outcomes from this review.

(1) Select the molecular component and the electrode material
  A homogeneous metal complex with optimal catalytic behavior is often a good starting point for a well-known reaction. Similarly, there is sometimes a previously employed combination with a specific electrode material. However, there are several things to keep in mind: (i) implementing a molecular handle for immobilization on the selected molecular catalyst may affect the catalytic behavior of the resulting system compared to the parent catalyst and (ii) successful immobilization does not necessarily result in an invariable translation of the reactivity of the homogeneous catalyst to the hybrid system.
(2) Evaluate and implement different immobilization strategies
  Learning from the examples discussed in Section 3, one should have an idea of how to immobilize a specific type of molecular catalyst onto an electrode surface. Depending on the reaction, and the tunability and stability of both components, a particular immobilization method may be more advantageous than another. However, immobilization does not guarantee an improved performance. The integration of two different types of catalysts to create a hybrid material renders the reaction complex. As a result, the interaction between the appended compound and the electrode shall be considered. The surface-molecule interactions not only depend on different linkage types (degree of conjugation), linkage length, and linkage flexibility but are also influenced by the local reaction environment. The impact of the immobilized electrode on catalytic activity and catalyst lifetime needs to be carefully evaluated under different conditions.
(3) Utilize different characterization methods to understand the hybrid system
  Cyclic voltammetry is a convenient tool to characterize redox-active surface-anchored molecular catalysts. However, a strong electrostatic interaction between the metal center and the electrode will limit the utility of cyclic voltammetry. Instead, cyclic voltammetry could produce confusing results, such as the lack of clear redox peaks. To address this issue, other characterization tools should be employed to assist surface analysis for the hybrid system, such as X-ray techniques that can help examine the existence of metal centers, their oxidation state, and their coordination environment.
4) Investigate catalytic activities in applications related to energy conversion
  It is common to observe more than one type of interaction in a hybrid system. Electronic and/or electrostatic interactions between the surface and the appended molecules may impact the catalytic activity. Furthermore, the catalytic behavior of the molecular catalyst does not always translate to the immobilized state. The catalytic activity of a hybrid system is a result of the combination of the parent molecular compound, the linkage used, and the reaction macroenvironment, including the solvent and the electrolyte. A comprehensive study of the molecule–surface interactions, electrode/electrolyte interface, electron transfer kinetics, and fundamental understanding of the reaction mechanism are necessary.^199^
5) Expand the reaction scope

Although the field of surface immobilization and electrode-supported molecular catalysis is growing, the majority of studies are concentrated on electrochemical transformations related to energy conversion. Electroorganic synthesis is an emerging field taking advantage of electricity to replace fossil fuel and/or toxic redox reagents; however, homogeneous electrocatalysts are almost exclusively employed.^3,159,189−198^ New electrode materials, represented by single-atom catalysts, molecularly modified electrodes, and other types of interfacial catalysts, are discussed only in a small number of reviews.^6,195,196^ Most importantly, the application of the electrode-immobilized catalysts is limited to products with only a few carbon atoms, in most cases, one or two; new electrode-supported catalysts should help produce more complicated structures than those currently produced, and even polymers.

**Figure 24:**
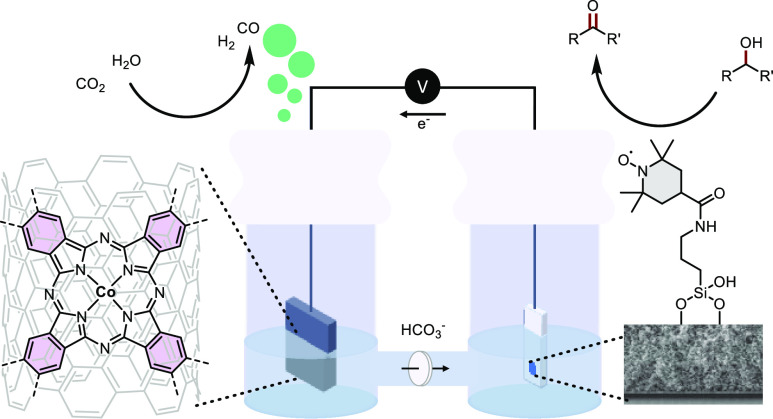
Integration of alcohol oxidation and CO_2_-to-syngas conversion in the same electrochemical setup.^39^ Adapted with permission from ref (39). Copyright 2020 John Wiley & Sons, Inc.

To bridge the gap between electrochemical small molecule activation such as CO_2_ reduction and electrochemical organic synthesis of valuable products with higher complexity, we suggest the following approaches:

(I) Design and develop a functionalized electrode
  Throughout this piece, we hope we demonstrated that, by immobilizing homogeneous catalysts, represented by metal complexes, the catalytic activity and selectivity of the tunable molecular structure can be integrated with recyclable and readily available electrode support. The molecularly modified electrode, which represents an emerging type of electrocatalyst, is worth investigating for future applications. The rational design and development of a hybrid catalyst could be optimized based on structure–property or structure–activity relationships of the active site, providing an extra degree of control over the catalytic activity.
(II) Investigate the potential of coupling redox events in both half cells
  From an energy efficiency point of view, it is important to emphasize coupling the events in both half cells. Although less emphasized in this review, it is preferable to conduct paired redox reactions for the full utilization of the cathode and anode.^39,200−203^ For example, a value-added process (i.e., CO_2_ to syngas) in the counter chamber can be coupled with a targeted reaction to reduce the energy loss during electrolysis (Figure 24).^39^ A similar strategy was applied using a bifunctional cobalt-modified electrode to achieve simultaneous CO_2_ reduction and water splitting.^201^ Other organic reactions also attempted paired electrolysis to conserve energy.^196,202^
(III) Integrate multistep transformations with multifunctional or interdisciplinary catalysts
  Upcycling of activated small molecules from electrochemical transformations such as CO_2_ reduction *via* a single-step process remains challenging in many cases.^204,205^ However, the use of multifunctional or several catalysts (homogeneous, heterogeneous, enzymatic, or photocatalyst) to conduct multistep transformations presents an attractive alternative.^200,203,206−211^ Compatibility, unfortunately, remains a major obstacle toward integrating multiple catalytic events in one system.^212^ Nonetheless, a polymerization catalyzed by a palladium catalyst was integrated with electrochemical CO_2_ reduction to CO, demonstrating the construction of polymeric material from a simple building block like CO_2_.^213^ Nonalternating polyketones with tunable CO content were obtained based on the applied current density.
(IV) Engineer the reaction setup

To implement the above-mentioned ideas and possibly advance some of the applications to industrial practice, a reaction setup that accommodates compatible, practical, and large-scale electrochemistry is needed. Limitations of the traditional design of electrochemical cells such as mass transportation impede its applications. High-throughput electrochemical cells, parallel plate cells, and flow reactors are designed to suit different needs and potentially address the issues in large-scale electrochemical synthesis.^6,13,159,193,196,207,214−220^

In summary, we highlight the potential of immobilizing molecular catalysts, especially organometallic catalysts, onto electrodes to afford a potent catalytic material to interconvert electrical and chemical energy. Such a hybrid system is constructed from a modified electrically addressable surface and metal complexes *via* a handful of immobilization methods. Effects on electron transfer rate, catalyst lifetime, activity, and product selectivity are discussed. Implications of functional groups, external electric fields, and solvents are emphasized for catalyst optimization. By presenting an array of applications implementing the organometallic complex modified electrode as the catalytically active material, we hope to convey the advantages of combining a homogeneous compound and a heterogeneous support in electrochemistry. We envision that the integration of present electrochemical activation of simple building blocks such as CO_2_ with subsequent processes represents a future direction of accomplishing complicated syntheses. Overall, the surface functionalization of molecular compounds on an electrically addressable surface heralds a new paradigm in designing and conducting chemical transformations.
